# Acid, silver, and solvent-free gold-catalyzed hydrophenoxylation of internal alkynes

**DOI:** 10.3762/bjoc.9.235

**Published:** 2013-10-02

**Authors:** Marcia E Richard, Daniel V Fraccica, Kevin J Garcia, Erica J Miller, Rosa M Ciccarelli, Erin C Holahan, Victoria L Resh, Aakash Shah, Peter M Findeis, Robert A Stockland Jr.

**Affiliations:** 1 Department of Chemistry, Bucknell University, Lewisburg PA, 17837, USA

**Keywords:** acid-free, catalysis, gold, gold catalysis, hydrophenoxylation, silver-free, single component, solvent-free

## Abstract

A range of arylgold compounds have been synthesized and investigated as single-component catalysts for the hydrophenoxylation of unactivated internal alkynes. Both carbene and phosphine-ligated compounds were screened as part of this work, and the most efficient catalysts contained either JohnPhos or IPr/SIPr. Phenols bearing either electron-withdrawing or electron-donating groups were efficiently added using these catalysts. No silver salts, acids, or solvents were needed for the catalysis, and either microwave or conventional heating afforded moderate to excellent yields of the vinyl ethers.

## Introduction

The use of gold compounds to promote hydroelementation reactions has grown tremendously over the past few years [1–5]. Using this approach, a host of different organic substrates have been successfully functionalized. Despite the intense interest in this chemistry the addition of phenols to internal alkenes has rarely been investigated [1,5]. Recently, Sahoo reported the hydrophenoxylation of alkynes using a multicomponent catalyst comprised of AuCl_3_, JohnPhos as the supporting ligand, and various silver/potassium salts as promoters [1]. He found that moderate to excellent yields of the vinyl ethers could be obtained upon heating the reaction mixtures (dichloromethane or THF solutions) to 100 °C for 24–146 hours. Given the current interest in the synthetic community regarding the development of sustainable transformations [6–10] that are practical and operationally straightforward, we decided to attempt to generate the active gold species in situ from a single-component precursor. Furthermore, one of the current goals of developing sustainable and “greener” organic reactions is focused on the elimination of solvents and additives from the synthetic methodology [10]. Thus, the design of gold catalyzed reactions that proceed under solvent-free conditions and without the addition of silver salts or acidic promoters would be of interest to those charged with designing sustainable organic reactions.

From the standpoint of the gold catalyst, there are several ways to generate an active species. One of the first reports on this type of catalysis entailed the use of strong acids to remove the methyl group from a methylgold compound and generate a (LAu^+^) species that promoted the addition reaction [11]. Additionally, these cationic gold species can be generated through the reaction of chlorogold compounds with silver salts [1–4]. Recently, there has been growing interest in developing approaches that generate a catalytically active species under acid-free and silver-free conditions [12–13]. To this end, we have synthesized and investigated a series of arylgold compounds as single-component catalysts for hydroelementation reactions under acid, silver, and solvent-free conditions.

**Scheme 1:**
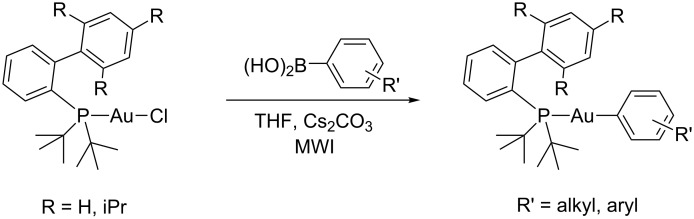
Microwave assisted synthesis of arylgold compounds.

We recently reported the synthesis of arylgold compounds using a focused microwave reactor (Scheme 1) [14]. Although microwave-assisted reactions are legion in organic chemistry, they are rare in organometallic synthesis [15–21]. Our initial report on this chemistry was focused on gold compounds bearing Buchwald-type phosphine ligands such as JohnPhos and *t*-BuXPhos. These bulky ligands were selected since they were found to inhibit dynamic phosphine exchange between gold complexes [22]. The chemistry was operationally straightforward, and simply heating the reagents in the microwave for 20–30 minutes afforded high yields of the arylgold compounds. In this report, we extend the chemistry to the synthesis of arylgold compounds containing carbene ligands.

## Results and Discussion

**Figure 1:**
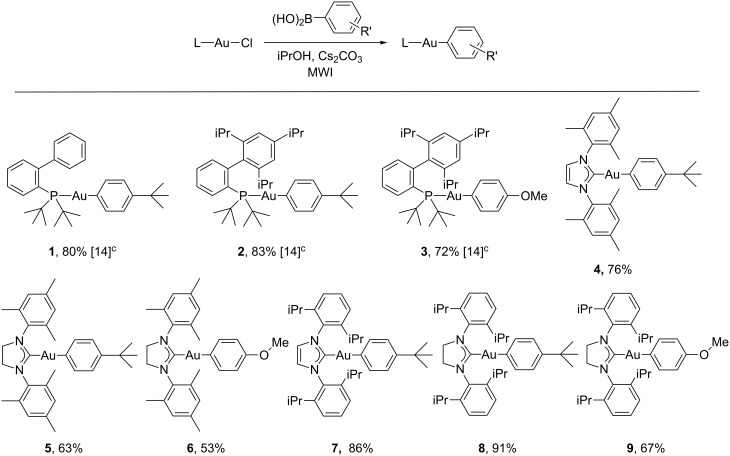
Synthesis of arylgold compounds^a,b^. ^a^Chlorogold precursor (0.32–0.37 mmol), 2 equiv arylboronic acid, 2 equiv Cs_2_CO_3_, 50 °C, 20 min, iPrOH (1.5 mL), microwave irradiation. ^b^Isolated yields. ^c^THF was used as the solvent.

The synthesis of the carbene-ligated arylgold compounds was accomplished using (NHC)AuCl (NHC = IMes, SIMes, IPr, SIPr) precursors. For the initial screening runs, (IMes)AuCl and 4-*tert*-butylphenylboronic acid were chosen as representative substrates. The same protocol that was successful for the synthesis of arylgold compounds from (JohnPhos)AuCl or (*t*-BuXPhos)AuCl afforded incomplete conversion and the formation of an intractable mixture of gold compounds when (IMes)AuCl was used. After some experimentation with the reaction conditions and different mineral bases, the key issue appeared to be the use of THF as the solvent. Changing from THF to iPrOH afforded high yields of (IMes)Au(C_6_H_4_*t*-Bu) after heating at 50 °C for 20 minutes. Once a successful protocol was determined for the model system, the chemistry was extended to other (NHC)AuCl compounds (Figure 1). It was anticipated that incorporating electron-donating groups into the aryl fragments on the gold would facilitate the cleavage of the organic group from the gold center through protodeauration; thus, –C_6_H_4_*t*-Bu and –C_6_H_4_OMe were selected as substituents. In general, the use of 4-*tert*-butylphenylboronic acid gave higher yields of the arylgold compounds than 4-methoxyphenylboronic acid. Once isolated by column chromatography (basic alumina), the arylgold compounds were relatively stable white solids. The IMes and SIMes examples (**4**–**6**) were the least stable of the group, and although no decomposition was noted when stored in the solid state under nitrogen (6 months) they begin to decompose within 24 hours in solution (THF). In contrast, the IPr and SIPr examples were quite stable in both solution and the solid state.

Once the arylgold compounds were isolated, the efficacy of both phosphine and carbene-ligated species in hydrophenoxylation reactions was investigated (Table 1). For these screening reactions, 4-nitrophenol, diphenylacetylene, and 5-decyne served as representative substrates. These alkynes were selected since unactivated internal alkynes remain some of the most challenging substrates to functionalize through hydroelementation reactions. Initially, we selected compound **1** as the gold catalyst and used a focused microwave reactor to heat the reactions. After some experimentation, we discovered that heating the phenol and alkyne with gold catalyst in the absence of solvent to 130 °C for 20 minutes generated excellent yields of the vinyl ethers. Control reactions revealed that the arylgold compound was essential to the success of the reaction as its removal lead to quantitative recovery of starting materials. The generation of the active gold catalyst in these reactions is proposed to occur through a protodeauration reaction between the arylgold compounds and the phenols. The observation of *tert*-butylbenzene in the reaction mixture supports this proposal. Furthermore, to probe whether or not simple chlorogold-coordination compounds could promote this reaction, JohnPhosAuCl and other gold compounds were investigated under the same reaction conditions (Table 1, entries 2–7). In all cases, the chlorogold compounds were unable to catalyze the addition reaction and analysis of the crude reaction mixtures revealed only starting materials. Conventional heating was also investigated during the screening phase of the project. Plunging a reactor vial containing **1**, 4-nitrophenol, and either internal alkyne into a preheated oil bath (130 °C, 20 min) followed by rapid cooling also afforded high yields of the vinyl ethers (Table 1, entry 18).

**Table 1:** Hydrophenoxylation of unactivated internal alkynes.^a^

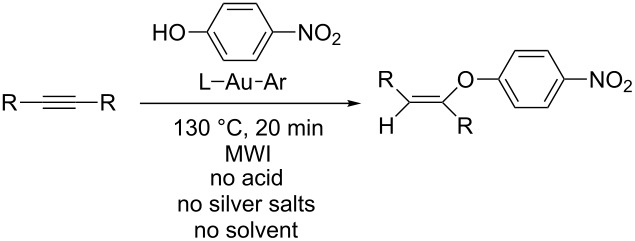			

Entry	Gold catalyst	Conv.^b^ R = Ph Bu	

1	None	0	0
2	(JohnPhos)AuCl	0	0
3	(*t*-BuXPhos)AuCl	0	0
4	(IMes)AuCl	0	0
5	(SIMes)AuCl	0	0
6	(IPr)AuCl	0	0
7	(SIPr)AuCl	0	0
8	**1**	98	95
9	**2**	62	48
10	**2** + *t*-BuXPhos	79	60
11	**3**	77	70
12	**4**	47	50
13	**5**	63	48
14	**6**	64	45
15	**7**	86	92
16	**8**	89	95
17	**9**	90	91
18	**1** Conventional heating	95	93

^a^Diphenylacetylene or 5-decyne (0.28 mmol), 4-nitrophenol (0.56 mmol), catalyst (14.0 μmol, 5%), 130 °C, 20 min, no solvent, microwave irradiation. ^b^Based upon ^1^H NMR spectroscopy using anisole as an internal standard.

The effectiveness of different gold catalysts on the addition reaction was also studied (Table 1). While **1** was effective at promoting the addition reaction, an arylgold compound bearing a bulkier Buchwald ligand (**2**) afforded lower yields of the vinyl ether. Adding an additional equivalent of *t*-BuXPhos increased the yield slightly; however, **1** was still a superior catalyst. Increasing the electron-donating ability of the substituent on the aromatic group attached to the gold center in the *t*-BuXPhos based catalyst **3** slightly increased the yield of the vinyl ether.

To investigate the effectiveness of carbene-ligated gold compounds on the addition reaction, catalysts **4**–**9** were screened (Table 1). When IMes or SIMes based catalysts **4**, **5**, or **6** were used, lower yields of the vinyl ethers were observed. Examining the reaction vessels when **4**, **5**, and **6** were used revealed the formation of a significant amount of metallic gold. These metallic deposits were not observed when using phosphine ligated gold compounds **1**–**3**. This observed decomposition/reduction to elemental gold could be a rationale for the lower conversions observed with these catalysts. Furthermore, changing to conventional heating did not prevent the decomposition of **4**, **5**, and **6** during the addition reaction. In contrast, the complexes bearing IPr and SIPr carbene ligands **7**–**9** afforded high yields of the vinyl ethers. Examination of the reaction vessels from these reactions did not reveal the formation of metallic gold. There was little difference between the saturated (SIPr) and unsaturated (IPr) carbene ligands, and there was only slight difference between catalysts bearing –C_6_H_4_*t*-Bu and –C_6_H_4_OMe aryl groups (**8**,**9**).

**Figure 2:**
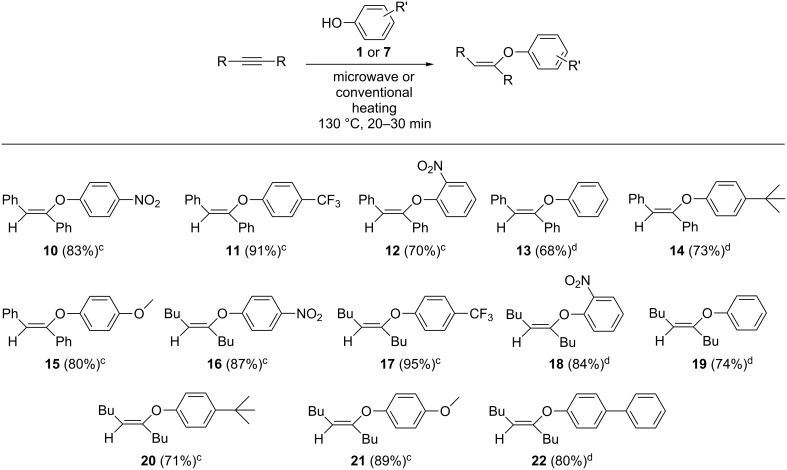
Hydrophenoxylation of alkynes^a,b^. ^a^Alkyne (0.28 mmol), phenol (0.56 mmol), 130 °C, 20 min, no solvent. ^b^Isolated yields. ^c^Microwave heating with **1** as the catalyst (14.0 μmol, 5%). ^d^Conventional heating with **7** as the catalyst (14.0 μmol, 5%).

Building on the results from the screening reactions, the scope of the hydrophenoxylation reaction was investigated using a range of phenols with diphenylacetylene and 5-decyne serving as representative internal alkynes (Figure 2). Microwave and conventional heating were used in these reactions. Based upon the initial studies, the phosphine ligated catalyst **1**, as well as NHC ligated catalysts **7**–**9** afforded high yields of the vinyl ethers. After a bit of experimentation, it was observed that **1** afforded the highest conversions in microwave-assisted reactions, while **7** generated the highest yields in conventionally heated reactions. The addition reaction was tolerant of both electron-withdrawing and electron-donating groups, and the ortho-substituted substrate 2-nitrophenol was also successfully used in the addition reactions. The resulting vinyl ethers were purified by column chromatography and isolated as powders or viscous oils.

**Scheme 2:**
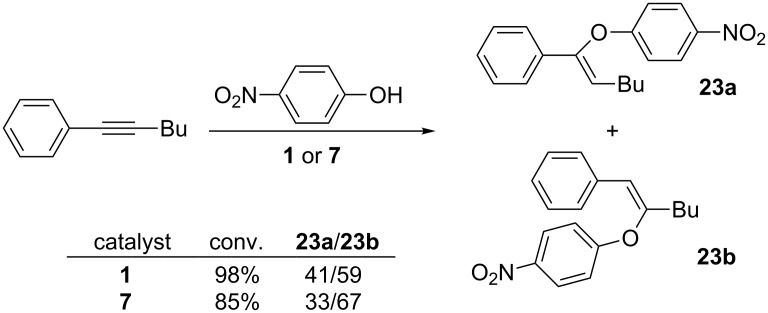
Regioselectivity of the addition reaction using arylgold precatalysts. Alkyne (0.28 mmol), phenol (0.56 mmol), 130 °C, 20 min, no solvent. Microwave heating with **1** or **7** as the catalyst (14.0 μmol, 5%). The yield of the addition product and ratio of regioisomers was determined by ^1^H NMR spectroscopy using anisole as an internal standard.

The regioselectivity of the addition reaction was investigated using 1-phenyl-1-hexyne as a representative unsymmetrical alkyne, 4-nitrophenol as the model phenol, and **1** and **7** as catalysts. As shown in Scheme 2, both catalysts generated the addition product in excellent yields. Catalyst **1** gave a slightly higher yield of the addition product, but **7** was more selective. The regioselectivity of the reaction using our arylgold precatalysts is the opposite of what Sahoo found using his catalyst system [1] and is similar to the regioselectivity found by Nolan using his hydroxide bridged precatalysts [5].

## Conclusion

In summary, we have synthesized several carbene-ligated arylgold compounds and investigated the efficacy of both phosphine and carbene-based gold catalysts towards the hydrophenoxylation of unactivated internal alkynes. Using these single component catalysts, a range of phenols have been successfully added to unactivated internal alkynes. No silver salts, acids, or solvents were needed for the catalysis, and either microwave or conventional heating and afforded moderate to excellent yields of the vinyl ethers.

## Experimental

**General considerations:** Unless specified, all solvents were dried using a Grubbs-type solvent purification system. (JohnPhos)AuCl and (*t*-BuXPhos)AuCl, were prepared by displacement of dimethyl sulfide from Me_2_SAuCl by JohnPhos or *t*-BuXPhos [23–24]. The (NHC)AuCl (NHC = SIMes, IMes, SIPr, IPr) [19,25] precursors as well as arylgold compounds **1**–**3** [14] were prepared following literature procedures. The arylboronic acids, alkynes, phenols, and Cs_2_CO_3_ (powder) were obtained from Aldrich and used as received. NMR spectra were collected on a Varian DirectDrive 600 MHz NMR spectrometer (^1^H: 599.77 MHz and ^13^C: 150.81 MHz) ^1^H and ^13^C{^1^H} chemical shifts were determined by reference to residual non-deuterated solvent resonances. The alkene geometry was determined using ^1^H–^1^H NOESY experiments. All coupling constants are listed in Hertz. Microwave-assisted reactions were carried out in sealed vessels using a CEM Discover equipped with an external IR (surface) temperature sensor. The PowerMax setting on the Discover was off. Conventionally heated reactions were carried out in an oil bath. HRMS data were obtained on a Thermo Scientific Exactive Plus LC–MS system (ESI).

**General method for the arylation reactions: general procedure A:** For a typical reaction, (NHC)AuCl, 2 equiv of the arylboronic acid, 2 equiv of Cs_2_CO_3_, and a magnetic stirring bar were added to a 10 mL reactor vial. After exchanging the air for nitrogen, isopropanol was added by syringe, the mixtures were triturated on a stirring plate for 3 minutes, and irradiated. The following settings were used for each experiment: Temperature = 50 °C, time = 20 min, initial power level = 25 W. The initial power setting listed for each reaction was maintained until the desired temperature was reached. The power was then reduced for the remainder of the reaction to maintain the temperature. The reaction time listed is the total irradiation time (no ramping periods). Note: High initial high levels of microwave power (to rapidly heat the sample) lead to significant decomposition (gold metal). After cooling to room temperature, the volatiles were removed, and the title compounds were purified by column chromatography (basic alumina). The arylgold compounds were dissolved in CH_2_Cl_2_ and dried over molecular sieves. After filtration, they were dried under vacuum to afford white powders.

**Preparation of (IMes)Au(4-C_6_H_4_*t*-Bu)** (**4**): General procedure A was followed with (IMes)AuCl (0.20 g, 0.37 mmol), 4-*tert*-butylphenylboronic acid (0.135 g, 0.76 mmol), cesium carbonate (0.24 g, 0.74 mmol), and isopropanol (1.5 mL). Chromatography: basic alumina (37.5 g), gradient hexane/THF (90:10–50:50), *R*_f_ 0.60 (hexane/THF 50:50), yield = 0.18 g of a white powder (76.1%). HRMS: [M + Na]^+^ calcd for C_31_H_37_AuN_2_Na, 657.2520; found, 657.2509. Spectral data: ^1^H NMR (CDCl_3_, 25 °C) δ 7.07 (s, 4H, Ar-H), 7.04 (s, 2H, =CH), 6.98 (br s, 4H, Ar-H), 2.34 (s, 6H, -Me), 2.16 (s, 12H, -Me), 1.19 (s, 9H, -Me); ^13^C{^1^H} NMR (CDCl_3_, 25 °C) δ 196.0 (s, carbene C), 165.4 (s, quat), 146.6 (s, quat), 139.9 (s, Ar-CH), 139.0 (s, quat), 135.4 (s, quat), 134.9 (s, quat), 129.2 (s, Ar-CH), 123.6 (s, Ar-CH), 121.7 (s, =CH), 34.0 (s, quat), 31.4 (s, -C*Me_3_*), 21.1 (s, -Me), 18.0 (s, -Me).

**General method for the catalyst screening reactions: microwave and conventional heating: general procedure B:** A reactor vial (10 mL) was charged with the LAuAr species (**1**–**9**, 0.014 mmol), alkyne (0.28 mmol), phenol (0.56 mmol), and a magnetic stirring bar. After exchanging the air for nitrogen, the samples were irradiated in a focused microwave reactor or heated in an oil bath. For the reactions carried out in the microwave reactor, the initial power setting listed for each reaction was maintained until the desired temperature was reached. No ramping periods were used in these reactions; thus, the reaction time listed is the total irradiation time (not the time at the desired temperature). After cooling, CDCl_3_ was added to the reaction mixtures until homogeneous solutions were obtained (≈2 mL). Anisole (internal standard, 0.28 mmol) was added to the solutions and the extent of each reaction was determined by ^1^H NMR spectroscopy.

**Isolation of the vinyl ethers:** The synthesis of the vinyl ethers was carried out following the same general procedure from the catalyst screening reactions using either **1** or **7** as the catalyst. Once cooled, the vinyl ethers were purified by column chromatography, dried using molecular sieves (hexane/EtOAc solution), and isolated as oils or powders following removal of the volatiles.

**Preparation of 4-[[(1*Z*)-1-butyl-1-hexen-1-yl]oxy]nitrobenzene (16):** General procedure B was followed (microwave heating) with **1** (0.0088 g, 0.014 mmol), 5-decyne (50.6 μL, 0.28 mmol), and 4-nitrophenol (0.078 g, 0.56 mmol). Temperature = 130 °C, time = 20 min, initial power level = 50 W. Chromatography: silica gel (19.1 g), gradient hexane/EtOAc (100:0–60:40). *R*_f_ 0.67 (hexane/EtOAc 95:5), yield = 0.068 g of a colorless oil (87%). HRMS: [M + H]^+^ calcd for C_16_H_24_NO_3_, 278.1758; found, 278.1748. Spectral data: ^1^H NMR (CDCl_3_, 25 °C) δ 8.19 (AA'BB', 2H, Ar-H), 6.99 (AA'BB', 2H, Ar-H), 5.12 (t, *J* = 7.2, 1H, =CH-), 2.15 (t, *J* = 7.8, 2H, -CH_2_-), 1.93 (q, *J* = 7.2, 2H, -CH_2_-), 1.47 (m, 2H, -CH_2_-), 1.34–1.25 (m, 6H, -CH_2_-), 0.89 (t, *J* = 7.2, 3H, -Me), 0.84 (t, *J* = 6.8, 3H, -Me); ^13^C{^1^H} NMR (CDCl_3_, 25 °C) δ 162.3 (s, quat), 150.0 (s, quat), 141.9 (s, quat), 126.0 (s, Ar-CH), 117.5 (s, =CH-), 115.5 (s, Ar-CH), 32.2 (s, -CH_2_-), 31.3 (s, -CH_2_-), 28.9 (s, -CH_2_-), 24.9 (s, -CH_2_-), 22.3 (s, -CH_2_-), 22.1 (s, -CH_2_-), 13.8 (s, -Me).

## Supporting Information

File 1 Detailed synthetic procedures for the synthesis of the arylgold compounds and vinyl ethers as well as NMR spectra for all new compounds.
