# Supplementary material for: Acid, silver, and solvent-free gold-catalyzed hydrophenoxylation of internal alkynes
Source: Beilstein J Org Chem. 2013 Oct 2;9:2002–8. doi: 10.3762/bjoc.9.235 (PMC3817533; doi:10.3762/bjoc.9.235)

Supporting Information

for

Acid, silver, and solvent-free gold catalyzed hydrophenoxylation of unactivated internal alkynes.

Marcia E. Richard, Daniel V. Fraccica, Kevin J. Garcia, Erica J. Miller, Rosa M. Ciccarelli, Erin C. Holahan, Victoria L. Resh, Aakash Shah, Peter M. Findeis and Robert A. Stockland Jr.*

Address: Department of Chemistry, Bucknell University, Lewisburg PA, 17837, USA

E-mail: Robert A. Stockland Jr.* - rstockla@bucknell.edu

* Corresponding author

Detailed synthetic procedures for the synthesis of the arylgold compounds and vinyl ethers as well as NMR spectra for all new compounds.

General considerations…………………………………………………………… S2

Synthesis and characterization of arylgold compounds…………………………. S2

Synthesis and characterization of vinyl ethers…………………………………… S6

References……………………………………………………………………………… S13

NMR spectra of new compounds…………………………………………………… S14

General considerations: Unless specified, all solvents were dried using a Grubbs-type solvent purification system. (JohnPhos)AuCl and (*t*-BuXPhos)AuCl, were prepared by displacement of dimethyl sulfide from Me_2SAuCl by JohnPhos or *t*-BuXPhos [1,2]. The (NHC)AuCl (NHC = SIMes, IMes, SIPr, IPr) [3,4] precursors as well as arylgold compounds **1**–**3** **[**5] were prepared following literature procedures. The arylboronic acids, alkynes, phenols, and Cs_2CO_3 (powder) were obtained from Aldrich and used as received. NMR spectra were collected on a Varian DirectDrive 600 MHz NMR spectrometer (^{1}H: 599.77 MHz and ^{13}C: 150.81 MHz). ^{1}H and ^{13}C{^{1}H} chemical shifts were determined by reference to residual non-deuterated solvent resonances. The alkene geometry was determined using ^{1}H–^{1}H NOESY experiments. All coupling constants are listed in Hertz. Microwave catalyzed reactions carried out in sealed vessels using a CEM Discover equipped with an external IR (surface) temperature sensor. The PowerMax setting on the Discover was off. Conventionally heated reactions were carried out in an oil bath**.** HRMS data were obtained on a Thermo Scientific Exactive Plus LC/MS system (ESI).

General method for the arylation reactions: general procedure A: For a typical reaction, (NHC)AuCl, 2 equiv of the arylboronic acid, 2 equiv of Cs_2CO_3, and a magnetic stirring bar were added to a 10 mL reactor vial. After exchanging the air for nitrogen, isopropanol was added by syringe. The mixtures were triturated on a stirring plate for 3 minutes followed by irradiation in the Discover microwave reactor. The following settings were used for each experiment: Temperature = 50 °C, time = 20 min, initial power level = 25 W. The initial power setting listed for each reaction was maintained until the desired temperature was reached. The power was then reduced for the remainder

of the reaction to maintain the temperature. The reaction time listed is the total irradiation time (no ramping periods). Note: High initial high levels of microwave power (to rapidly heat the sample) resulted in significant decomposition (gold metal). After cooling to room temperature, the volatiles were removed, and the title compounds were purified by column chromatography (basic alumina). The arylgold compounds were dissolved in CH_2Cl_2 and dried over molecular sieves. After filtration, they were dried under vacuum to afford white powders.

Preparation of (IMes)Au(4-C_6H_4*t*-Bu) (4). General procedure A was followed with (IMes)AuCl (0.20 g, 0.37 mmol), 4-*tert*-butylphenylboronic acid (0.135 g, 0.76 mmol), cesium carbonate (0.24 g, 0.74 mmol), and isopropanol (1.5 mL). Chromatography: basic alumina (37.5 g), gradient hexane/THF (90:10–50:50). R_f 0.60 (hexane/THF 50:50), yield = 0.18 g of a white powder (76.1%). HRMS: $[M + Na]^+$ calcd for $C_{31}H_{37}AuN_2Na$: 657.2520; found, 657.2509. Spectral data: ^1H NMR ($CDCl_3$, 25 °C) δ 7.07 (s, 4H, Ar-H), 7.04 (s, 2H, =CH), 6.98 (br s, 4H, Ar-H), 2.34 (s, 6H, -Me), 2.16 (s, 12H, -Me), 1.19 (s, 9H, -Me). $^{13}C\{^1H\}$ NMR ($CDCl_3$, 25 °C) δ 196.0 (s, carbene C), 165.4 (s, quat), 146.6 (s, quat), 139.9 (s, Ar-CH), 139.0 (s, quat), 135.4 (s, quat), 134.9 (s, quat), 129.2 (s, Ar-CH), 123.6 (s, Ar-CH), 121.7 (s, =CH), 34.0 (s, quat), 31.4 (s, -CMe_3), 21.1 (s, -Me), 18.0 (s, -Me).

Preparation of (SIMes)Au(4-C_6H_4*t*-Bu) (5). General procedure A was followed with (SIMes)AuCl (0.20 g, 0.37 mmol), 4-*tert*-butylphenylboronic acid (0.135 g, 0.76 mmol), cesium carbonate (0.24 g, 0.74 mmol), and isopropanol (1.5 mL). Chromatography: basic

alumina (37.5 g), gradient hexane/THF (90:10–50:50). R_f 0.62 (hexane/THF 50:50), yield = 0.15 g of a white powder (63.5%). HRMS: $[M + Na]^+$ calcd for $C_{31}H_{39}AuN_2Na$: 659.2676; found, 659.2683. Spectral data: ^1H ($CDCl_3$, 25 °C) δ 7.00 (AA′BB′, 4H, Ar-H), 6.94 (s, 4H, Ar-H), 3.93 (s, 4H, $-NCH_2-$), 2.37 (s, 12H, -Me), 2.30 (s, 6H, -Me), 1.18 (s, 9H, -Me). $^{13}C\{^1H\}$ NMR ($CDCl_3$, 25 °C) δ 216.4 (s, carbene C), 165.7 (s, quat), 146.7 (s, quat), 139.9 (s, Ar-CH), 138.2 (s, quat), 135.8 (s, quat), 135.4 (s, quat), 129.4 (s, Ar-CH), 123.5 (s, Ar-CH), 50.8 (s, $-NCH_2-$), 34.0 (s, quat), 31.4 (s, -Me), 21.1 (s, -Me), 18.1 (s, -Me).

Preparation of (SIMes)Au(4-C_6H_4OMe) (6). General procedure A was followed with (SIMes)AuCl (0.20 g, 0.37 mmol), 4-methoxyphenylboronic acid (0.115 g, 0.76 mmol), cesium carbonate (0.24 g, 0.74 mmol), and isopropanol (1.5 mL). Chromatography: basic alumina (37.5 g), gradient hexane/THF (90:10–50:50). R_f 0.45 (hexane/THF 50:50), yield = 0.12 g of a white powder (53.0%). HRMS: $[M + Na]^+$ calcd for $C_{28}H_{33}AuN_2ONa$: 633.2156; found, 633.2144. Spectral data: ^1H NMR ($CDCl_3$, 25 °C) δ 6.95 (s, 4H, Ar-H), 6.95 (AA'BB', 2H, Ar-H), 6.63 (AA'BB', 2H, Ar-H), 3.93 (s, 3H, $-NCH_2-$), 3.66 (s, 3H, -OMe), 2.37 (s, 12H, -Me), 2.31 (s, 6H, -Me). $^{13}C\{^1H\}$ NMR ($CDCl_3$, 25 °C) δ 216.2 (s, carbene C), 160.3 (s, quat), 157.1 (s, quat), 140.7 (s, Ar-CH), 138.2 (s, quat), 135.8 (s, quat), 135.4 (s, quat), 129.4 (s, Ar-CH), 112.7 (s, Ar-CH), 55.0 (s, -OMe), 50.9 (s, $-NCH_2-$), 21.1 (s, -Me), 18.2 (s, -Me).

Preparation of (IPr)Au(4-C_6H_4*t*-Bu) (7). General procedure A was followed with (IPr)AuCl (0.20 g, 0.32 mmol), 4-*tert*-butylphenylboronic acid (0.115 g, 0.65 mmol),

cesium carbonate (0.21 g, 0.64 mmol), and isopropanol (1.5 mL). Chromatography: basic alumina (37.5 g), gradient hexane/THF (90:10-50:50). R_f 0.69 (hexane/THF 50:50), yield = 0.20 g of a white powder (86.4%). HRMS: $[M + Na]^+$ calcd for $C_{37}H_{49}AuN_2Na$: 741.3459; found, 741.3418. Spectral data: ^{1}H NMR ($CDCl_3$, 25 °C) δ 7.44 (t, 2H, *J* = 7.5, Ar-H), 7.25 (d, 4H, *J* = 7.2, Ar-H), 7.13 (s, 2H, =CH-), 7.05 (s, 4H, Ar-H), 2.67 (sept, 4H, *J* = 6.9, iPrCH), 1.41 (d, 12H, *J* = 7.2, iPr-Me), 1.23 (d, 12H, *J* = 7.2, iPr-Me), 1.18 (s, 9H, -Me). ^{13}C{^{1}H} NMR ($CDCl_3$, 25 °C) δ 197.3 (s, carbene C), 166.1 (s, quat), 146.2 (s, quat), 145.7 (s, quat), 140.0 (s, Ar-CH), 134.7 (s, quat), 130.1 (s, Ar-CH), 123.9 (s, Ar-CH), 123.5 (s, Ar-CH), 122.7 (s, =CH-), 34.0 (s, -*C*Me_3), 31.4 (s, -C*Me_3*), 28.8 (s, iPr-CH), 24.5 (s, iPr-Me), 23.9 (s, iPr-Me).

Preparation of (SIPr)Au(4-C_6H_4*t*-Bu) (8). General procedure A was followed with (SIPr)AuCl (0.20 g, 0.32 mmol), 4-*tert*-butylphenylboronic acid (0.115 g, 0.65 mmol), cesium carbonate (0.21 g, 0.64 mmol), and isopropanol (1.5 mL). Chromatography: basic alumina (37.5 g), gradient hexane/THF (90:10-50:50). R_f 0.70 (hexane/THF 50:50), yield = 0.21 g of a white powder (91%). HRMS: $[M + Na]^+$ calcd for $C_{37}H_{51}AuN_2Na$: 743.3618; found, 743.3574. Spectral data: ^{1}H NMR ($CDCl_3$, 25 °C) δ 7.34 (t, 2H, *J* = 7.5, Ar-H), 7.20 (d, 4H, *J* = 8.4, Ar-H), 7.02 (AA'BB', 2H, Ar-H), 6.96 (AA'BB', 2H, Ar-H), 3.98 (s, 4H, -NCH_2-), 3.15 (sept, 4H, *J* = 6.8, iPr-CH), 1.48 (d, 12H, *J* = 7.2, iPr-Me), 1.34 (d, 12H, *J* = 7.2, iPr-Me), 1.16 (s, 9H, -CMe_3). ^{13}C{^{1}H} NMR ($CDCl_3$, 25 °C) δ 216.7 (s, carbene C), 166.4 (s, quat), 146.7 (s, quat), 146.1 (s, quat), 139.9 (s, Ar-CH), 134.8 (s, quat), 129.4 (s, Ar-CH), 124.3 (s, Ar-CH), 123.4 (s, Ar-CH), 53.7 (s, -NCH_2-), 34.0 (s, -*C*Me_3), 31.4 (s, -C*Me_3*), 29.0 (s, iPr-CH), 25.1 (s, iPr-Me), 24.0 (s, iPr-Me).

Preparation of (SIPr)Au(4-C_6H_4OMe) (9). General procedure A was followed with (SIPr)AuCl (0.20 g, 0.32 mmol), 4-methoxyphenylboronic acid (0.10 g, 0.66 mmol), cesium carbonate (0.21 g, 0.64 mmol), and isopropanol (1.5 mL). Chromatography: basic alumina (37.5 g), gradient hexane/THF (90:10–50:50). R_f 0.65 (hexane/THF 50:50), yield = 0.15 g of a white powder (67.3%). HRMS: $[M + Na]^+$ calcd for $C_{34}H_{45}AuN_2ONa$: 717.3098; found, 717.3055. Spectral data: ^{1}H NMR ($CDCl_3$, 25 °C) δ 7.36 (t, 2H, J = 7.8, Ar-H), 7.21 (d, 4H, J = 7.8, Ar-H), 6.92 (d, 2H, J = 8.4, Ar-H), 6.60 (d, 2H, J = 8.4, Ar-H), 4.00 (s, 4H, -NCH_2-), 3.63 (s, 3H, -OMe), 3.15 (sept, 4H, J = 6.9, iPr-CH), 1.47 (d, 12H, J = 7.2, iPr-Me), 1.35 (d, 12H, J = 7.2, iPr-Me). ^{13}C{^{1}H} NMR ($CDCl_3$, 25 °C) δ 216.6 (s, carbene C), 160.9 (s, quat), 156.8 (s, quat), 146.7 (s, quat), 140.6 (s, Ar-CH), 134.8 (s, quat), 129.4 (s, Ar-CH), 124.3 (s, Ar-CH), 112.6 (s, Ar-CH), 54.9 (s, -NCH_2-), 53.7 (s, -OMe), 29.0 (s, iPr-CH), 25.1 (s, iPr-Me), 24.1 (s, iPr-Me).

General method for the catalyst screening reactions: microwave and conventional heating: general procedure B: A reactor vial (10 mL) was charged with the LAuAr species (**1**–**9**, 0.014 mmol), alkyne (0.28 mmol), phenol (0.56 mmol), and a magnetic stirring bar. After exchanging the air for nitrogen, the samples were irradiated in a focused microwave reactor or heated in an oil bath. For the reactions carried out in the microwave reactor, the initial power setting listed for each reaction was maintained until the desired temperature was reached. No ramping periods were used in these reactions; thus, the reaction time listed is the total irradiation time (not the time at the desired temperature). After cooling, $CDCl_3$ was added to the reaction mixtures until

homogeneous solutions were obtained (≈2 mL). Anisole (internal standard, 0.28 mmol) was added to the solutions and the extent of each reaction was determined by ^{1}H NMR spectroscopy.

Isolation of the vinyl ethers: The synthesis of the vinyl ethers was carried out following the same general procedure from the catalyst screening reactions using either **1** or **7** as the catalyst. Once cooled, the vinyl ethers were purified by column chromatography, dried using molecular sieves (hexane/EtOAc solution), and isolated as oils or powders following removal of the volatiles.

Preparation of 1-[[(1*Z*)-1,2-diphenylethenyl]oxy]-4-nitrobenzene (10), CAS: 1219621-28-9 [6]. General procedure B was followed (microwave heating) using **1** (0.0088 g, 0.014 mmol), diphenylacetylene (0.050 g, 0.28 mmol), and 4-nitrophenol (0.078 g, 0.56 mmol). Temperature = 130 °C, time = 20 min, initial power level = 50 W. Chromatography: silica gel (19.1 g), hexane/ETOAc (90:10). R_f 0.62 (hexane/EtOAc 90:10), yield = 0.074 g of a white powder (83%).

Preparation of 1-[[(1*Z*)-1,2-diphenylethenyl]oxy]-4-trifluoromethylbenzene (11). CAS: 1219621-27-8 [6]. General procedure B was followed (microwave heating) with **1** (0.0088 g, 0.014 mmol), diphenylacetylene (0.050 g, 0.28 mmol), and 4-(trifluoromethyl)phenol (0.091 g, 0.56 mmol). Temperature = 130 °C, time = 20 min, initial power level = 50 W. Chromatography: silica gel (19.1 g), hexane/EtOAc (95:5). R_f 0.56 (hexane/EtOAc 95:5), yield = 0.086 g of a white powder (91%).

Preparation of 1-[[(1*Z*)-1,2-diphenylethenyl]oxy]-2-nitrobenzene (12), CAS: 1219621-36-9 [6]. General procedure B was followed (microwave heating) with **1** (0.0088 g, 0.014 mmol), diphenylacetylene (0.050 g, 0.28 mmol), and 2-nitrophenol (0.078 g, 0.56 mmol). Temperature = 130 °C, time = 20 min, initial power level = 50 W. Chromatography: silica gel (19.1 g), hexane/EtOAc (95:5). R_f 0.40 (hexane/EtOAc 95:5), yield = 0.062 g of a pale yellow powder (70%).

Preparation of 1-[[(1*Z*)-1,2-diphenylethenyl]oxy]benzene (13), CAS: 1219621-30-3 [6]. General procedure B was followed (conventional heating) with **7** (0.010 g, 0.014 mmol) diphenylacetylene (0.050 g, 0.28 mmol)), and phenol (0.053 g, 0.56 mmol). Temperature = 130 °C, time = 20 min. Chromatography: silica gel (19.1 g), hexane/EtOAc (93:7). R_f 0.74, hexane/EtOAc (93:7), yield = 0.052 g of a white powder (68%).

Preparation of 1-[[(1*Z*)-1,2-diphenylethenyl]oxy]-4-*tert*-butylbenzene (14). General procedure B was followed (conventional heating) with **7** (0.010 g, 0.014 mmol), diphenylacetylene (0.050 g, 0.28 mmol), and 4-*tert*-butylphenol (0.084 g, 0.56 mmol). Temperature = 130 °C, time = 20 min. Chromatography: silica gel (19.1 g), gradient hexane/EtOAc (100:0–98:2). R_f 0.41 (hexane/EtOAc 98:2), yield = 0.067 g of a white powder (73%). HRMS: $[M + H]^+$ calcd for $C_{24}H_{25}O$: 329.1907; found, 329.1903. Spectral data: ^1H NMR ($CDCl_3$, 25 °C) δ 7.64 (d, 2H, *J* = 7.8, Ar-H), 7.59 (d, 2H, *J* = 7.2, Ar-H), 7.31–7.17 (m, 8H, Ar-H), 6.93 (AA'BB', 2H, Ar-H), 6.62 (s, 1H, =CH-), 1.23 (s, 9H, -C*Me*$_3$). $^{13}C\{^1H\}$ NMR ($CDCl_3$, 25 °C) δ 154.0 (s, quat), 149.9 (s, quat), 144.5 (s, quat),

136.3 (s, quat), 134.9 (s, quat), 129.0 (s, Ar-CH), 128.52 (s, Ar-CH), 128.50 (s, Ar-CH), 128.3 (s, Ar-CH), 127.3(s, Ar-CH), 126.4 (s, Ar-CH), 126.1 (s, Ar-CH), 116.7 (s, =CH-), 115.6 (s, Ar-CH), 34.1 (s, -*C*Me$_3$), 31.5 (s, -C*Me$_3$*).

Preparation of 1-[[(1*Z*)-1,2-diphenylethenyl]oxy]-4-methoxybenzene (15), CAS: 1219621-22-3 [6]. General procedure B was followed (microwave heating) with **1** (0.0088 g, 0.014 mmol), diphenylacetylene (0.050 g, 0.28 mmol), and 4-methoxyphenol (0.070 g, 0.56 mmol). Temperature = 130 °C, time = 20 min, initial power level = 50 W. Chromatography: silica gel (19.1 g), hexane/EtOAc (100:0–90:10). R_f 0.51 (hexane/EtOAc 95:5), yield = 0.068 g of a white powder (80%).

Preparation of 4-[[(1*Z*)-1-butyl-1-hexen-1-yl]oxy]nitrobenzene (16). General procedure B was followed (microwave heating) with **1** (0.0088 g, 0.014 mmol), 5-decyne (50.6 μL, 0.28 mmol), and 4-nitrophenol (0.078 g, 0.56 mmol). Temperature = 130 °C, time = 20 min, initial power level = 50 W. Chromatography: silica gel (19.1 g), gradient hexane/EtOAc (100:0–60:40). R_f 0.67 (hexane/EtOAc 95:5), yield = 0.068 g of a colorless oil (87%). HRMS: $[M + H]^+$ calcd for $C_{16}H_{24}NO_3$: 278.1758; found, 278.1748. Spectral data: ^{1}H NMR ($CDCl_3$, 25 °C) δ 8.19 (AA'BB', 2H, Ar-H), 6.99 (AA'BB', 2H, Ar-H), 5.12 (t, 1H, *J* = 7.2, =CH-), 2.15 (t, 2H, *J* = 7.8, -CH$_2$-), 1.93 (q, 2H, *J* = 7.2 -CH$_2$-), 1.47 (m, 2H, -CH$_2$-), 1.34–1.25 (m, 6H, -CH$_2$-), 0.89 (t, 3H, *J* = 7.2, -Me), 0.84 (t, 3H, *J* = 6.8, -Me). $^{13}C\{^1H\}$ NMR ($CDCl_3$, 25 °C) δ 162.3 (s, quat), 150.0 (s, quat), 141.9 (s, quat), 126.0 (s, Ar-CH), 117.5 (s, =CH-), 115.5 (s, Ar-CH), 32.2 (s, -CH$_2$-), 31.3 (s, -CH$_2$-), 28.9 (s, -CH$_2$-), 24.9 (s, -CH$_2$-), 22.3 (s, -CH$_2$-), 22.1 (s, -CH$_2$-), 13.8 (s, -CH$_3$).

Preparation of [[(1*Z*)-1-butyl-1-hexen-1-yl]oxy]-4-(trifluoromethyl)benzene (17). General procedure B was followed (microwave heating) with **1** (0.0088 g, 0.014 mmol), 5-decyne (50.6 μL, 0.28 mmol), and 4-(trifloromethyl)phenol (0.091 g, 0.56 mmol). Temperature = 130 °C, time = 20 min, initial power level = 50 W. Chromatography: silica gel (19.1 g), hexane. R_f 0.86 (hexane), yield = 0.080 g of a colorless oil (95%). HRMS: $[M + H]^+$ calcd for $C_{17}H_{24}F_3O$: 301.1781; found, 301.1771. Spectral data: ^1H NMR ($CDCl_3$, 25 °C) δ 7.53 (d, 2H, J = 7.8, Ar-H), 6.98 (d, 2H, J = 8.4, Ar-H), 5.07 (t, 1H, J = 7.2, =CH-), 2.12 (t, 2H, J= 7.5, $-CH_2-$), 1.96 (q, 2H, J = 7.2, $-CH_2-$), 1.44 (m, 2H, $-CH_2-$), 1.35-1.25 (m, 6H, $-CH_2-$), 0.88 (t, 3H, J= 7.2, -Me), 0.85 (t, 3H, J = 7.2, -Me). $^{13}C\{^1H\}$ NMR ($CDCl_3$, 25 °C) δ 159.5 (s, quat), 150.1 (s, quat), 126.9 (q, J = 3.9, Ar-CH), 124.4 (q, J= 271.3, -CF_3), 123.3 (q, J = 32.4, quat), 117.0 (s, =CH-), 115.6 (s, Ar-CH), 32.0 (s, $-CH_2-$), 31.5 (s, $-CH_2-$), 29.0 (s, $-CH_2-$), 24.9 (s, $-CH_2-$), 22.3 (s, $-CH_2-$), 22.1 (s, $-CH_2-$), 13.8 (s, -Me).

Preparation of 2-[[(1*Z*)-1-butyl-1-hexen-1-yl]oxy]nitrobenzene (18). General procedure B was followed (conventional heating) with **7** (0.010 g, 0.014 mmol), 5-decyne (50.6 μL, 0.28 mmol), and 2-nitrophenol (0.078 g, 0.56 mmol). Temperature = 130 °C, time = 30 min. Chromatography: basic alumina (27.5 g), gradient hexane/THF (100:0–95:5). R_f 0.80 (hexane/THF 95:5), yield = 0.066 g of a pale yellow oil (84%). HRMS: $[M + H]^+$ calcd for $C_{16}H_{24}NO_3$: 278.1758 ; found, 278.1747. Spectral data: ^1H NMR ($CDCl_3$, 25 °C) δ 7.87 (d, 1H, J = 7.0, Ar-H), 7.46 (t, 1H, J = 8.1, Ar-H), 7.04 (d, 1H, J = 7.2, Ar-H), 7.03 (t, 1H, J = 7.8, Ar-H), 5.12 (t, 1H, J = 6.9, =CH-), 2.15 (t, 2H, J = 7.8,

-CH_2-), 1.98 (q, 2H, *J* = 7.2, -CH_2-), 1.47 (m, 2H, -CH_2-), 1.37 - 1.26 (m, 6H, -CH_2-), 0.88 (t, 3H, *J* = 7.2, -Me), 0.85 (t, 3H, *J* = 6.9, -Me). $^{13}C\{^1H\}$ NMR ($CDCl_3$, 25 °C) δ 150.3 (s, quat), 149.8 (s, quat), 139.7 (s, quat), 133.8 (s, Ar-CH), 125.7 (s, Ar-CH), 120.8 (s, Ar-CH), 117.5 (s, =CH-), 116.3 (s, Ar-CH), 32.0 (s, -CH_2-), 31.3 (s, -CH_2-), 28.8 (s, -CH_2-), 24.8 (s, -CH_2-), 22.2 (s, -CH_2-), 22.1 (s, -CH_2-), 13.81 (s, -Me), 13.79 (s, -Me).

Preparation of [[(1*Z*)-1-butyl-1-hexen-1-yl]oxy]benzene (19). General procedure B was followed (conventional heating) with **7** (0.010 g, 0.014 mmol), 5-decyne (50.6 μL, 0.28 mmol), and phenol (0.053 g, 0.56 mmol). Temperature = 130 °C, time = 20 min. Chromatography: silica gel (19.1 g), hexane. R_f 0.71 (hexane), yield = 0.048 g of a colorless oil (74%). HRMS: $[M + H]^+$ calcd for $C_{16}H_{25}O$: 233.1907; found, 233.1898. Spectral data: ^1H NMR ($CDCl_3$, 25 °C) δ 7.26 (t, 2H, *J* = 8.4, Ar-H), 6.95 (t, 1H, *J* = 7.5, Ar-H), 6.92 (d, 2H, *J* = 7.5, Ar-H), 5.00 (t, 1H, *J* = 7.2, =CH-), 2.11 (t, 2H, *J* = 7.2, -CH_2-), 2.01 (q, 2H, *J* = 7.2, -CH_2-), 1.43 (m, 2H, -CH_2-), 1.29 (m, 6H, -CH_2-), 0.87 (t, 3H, *J* = 7.2, -CH_3), 0.85 (t, 3H, *J* = 7.2, -CH_3). $^{13}C\{^1H\}$ NMR ($CDCl_3$, 25 °C) δ 156.8 (s, quat), 150.6 (s, quat), 129.4 (s, Ar-CH), 121.2 (s, Ar-CH), 116.1 (s, =CH-), 115.9 (s, Ar-CH), 32.0 (s, -CH_2-), 31.7 (s, -CH_2-), 29.1 (s, -CH_2-), 24.8 (s, -CH_2-), 22.3 (s, -CH_2-), 22.2 (s, -CH_2-), 13.90 (s, -Me), 13.87 (s, -Me).

Preparation of [[(1*Z*)-1-butyl-1-hexen-1-yl]oxy]-4-*tert*-butylbenzene (20). General procedure B was followed (microwave heating) with **1** (0.0088 g, 0.014 mmol), 5-decyne (50.6 μL, 0.28 mmol), and 4-*tert*-butylphenol (0.084 g, 0.56 mmol). Temperature = 130 °C, time = 20 min, initial power level = 50 W. Chromatography: silica gel (19.1 g),

hexane. R_f 0.53 (hexane), yield = 0.057 g of a colorless oil (71%). HRMS: $[M + H]^+$ calcd for $C_{20}H_{33}O$: 289.2533; found, 289.2524. Spectral data: ^1H NMR ($CDCl_3$, 25 °C) δ 7.27 (AA'BB', 2H, Ar-H), 6.84 (AA'BB', 2H, Ar-H), 4.98 (t, 1H, *J* = 7.2, =CH-), 2.10 (t, 2H, *J* = 7.5, $-CH_2-$), 2.02 (q, 2H, *J* = 7.0, $-CH_2-$), 1.43 (m, 2H, $-CH_2-$), 1.34-1.26 (m, 6H, $-CH_2-$), 1.30 (s, 9H, -C*Me₃*), 0.87 (t, 3H, *J* = 7.5, $-CH_3$), 0.85 (t, 3H, *J* = 6.9, $-CH_3$). $^{13}C\{^1H\}$ NMR ($CDCl_3$, 25 °C) δ 154.4 (s, quat), 150.8 (s, quat), 143.9 (s, quat), 126.2 (s, Ar-CH), 115.9 (s, =CH-), 115.4 (s, Ar-CH), 34.1 (s, -*C*Me_3), 32.0 (s, $-CH_2-$), 31.8 (s, $-CH_2-$), 31.6 (s, -CMe_3), 29.1 (s, $-CH_2-$), 24.9 (s, $-CH_2-$), 22.4 (s, $-CH_2-$), 22.2 (s, $-CH_2-$), 14.0 (s, -Me), 13.9 (s, -Me).

Preparation of [[(1*Z*)-1-butyl-1-hexen-1-yl]oxy]-4-methoxybenzene (21). General procedure B was followed (microwave heating) with **1** (0.0088 g, 0.014 mmol), 5-decyne (50.6 μL, 0.28 mmol), and 4-methoxyphenol (0.070 g, 0.56 mmol). Temperature = 130 °C, time = 20 min, initial power level = 50 W. Chromatography: silica gel (19.1 g), hexane/EtOAc (95:5). R_f 0.86 hexane/EtOAc (95:5), yield = 0.066 g of a colorless oil (89%). HRMS: $[M + H]^+$ calcd for $C_{17}H_{27}O_2$: 262.2013; found, 263.2011. Spectral data: ^1H NMR ($CDCl_3$, 25 °C) δ 6.85 (AA'BB', 2H, Ar-H), 6.81 (AA'BB', 2H, Ar-H), 4.94 (t, 1H, *J* = 6.9, =CH-), 3.78 (s, 3H, -OMe), 2.07 (t, 2H, *J* = 7.5, $-CH_2-$), 2.04 (q, 2H, *J* = 7.0, $-CH_2-$), 1.41 (m, 2H, $-CH_2-$), 1.30 (m, 6H, $-CH_2-$), 0.86 (t, 6H, *J* = 7.2, -Me). $^{13}C\{^1H\}$ NMR ($CDCl_3$, 25 °C) δ 154.3 (s, quat), 151.1 (s, quat), 150.6 (s, quat), 117.0 (s, Ar-CH), 115.4 (s, =CH-), 114.6 (s, Ar-CH), 55.7 (s, -OMe), 31.8 (s, $-CH_2-$), 29.1 (s, $-CH_2-$), 24.8 (s, $-CH_2-$), 22.4 (s, $-CH_2-$), 22.1 (s, $-CH_2-$), 13.92 (s, -Me), 13.87 (s, -Me).

Preparation of [[(1*Z*)-4-[1-butyl-1-hexen-1-yl]oxy]]-1,1'-biphenyl (22). General procedure B was followed (conventional heating) with **7** (0.010 g, 0.014 mmol), 5-decyne (50.6 μL, 0.28 mmol), and 4-phenylphenol (0.096 g, 56 mmol). Temperature = 130 °C, time = 20 min. Chromatography: silica gel (19.1 g), gradient hexane/EtOAc (100:0–95:5). R_f 0.81 (hexane/EtOAc 95:5), yield = 0.069 g of a colorless oil (80%). HRMS: $[M + H]^+$ calcd for $C_{22}H_{29}O$: 309.2220; found, 309.2211. Spectral data: ^1H NMR ($CDCl_3$, 25 °C) δ 7.54 (d, 2H, *J* = 7.2, Ar-H), 7.50 (AA'BB', 2H, Ar-H), 7.40 (t, 2H, *J* = 7.5, Ar-H), 7.29 (t, 1H, *J* = 7.5, Ar-H), 6.98 (AA'BB', 2H, Ar-H), 5.03 (t, 1H, *J* = 6.9, =CH), 2.15 (t, 2H, *J* = 7.2, $-CH_2-$), 2.04 (q, 2H, *J* = 7.2, $-CH_2-$), 1.46 (m, 2H, $-CH_2-$), 1.36–1.26 (m, 6H, $-CH_2-$), 0.88 (t, 3H, *J* = 6.9, -Me), 0.87 (t, 3H, *J* = 7.2, -Me). $^{13}C\{^1H\}$ NMR ($CDCl_3$, 25 °C) δ 156.4 (s, quat), 150.6 (s, quat), 140.8 (s, quat), 134.4 (s, quat), 128.7 (s, Ar-CH), 128.2 (s, Ar-CH), 126.8 (s, Ar-CH), 126.7 (s, Ar-CH), 116.3 (s, =CH-), 116.2 (s, Ar-CH), 32.1 (s, $-CH_2-$), 31.7 (s, $-CH_2-$), 29.1 (s, $-CH_2-$), 24.9 (s, $-CH_2-$), 22.4 (s, $-CH_2-$), 22.2 (s, $-CH_2-$), 13.94 (s, $-CH_3$), 13.91 (s, $-CH_3$).

References

1. Nieto-Oberhuber, C.; Lopez, S.; Echavarren, A. M. *J. Am. Chem. Soc.* **2005**, *127*, 6178-6179. doi:10.1021/ja042257t
2. Hashmi, A. S. K.; Ata, F.; Haufe, P.; Rominger, F. *Tetrahedron* **2009**, *65*, 1919-1927. doi:10.1016/j.tet.2008.12.058
3. Landers, B.; Navarro, O. *Eur. J. Inorg. Chem.* **2012**, 2980-2982. doi:10.1002/ejic.201200248
4. de Fremont, P.; Scott, N. M.; Stevens, E. D.; Nolan, S. P. Organometallics 2005, 24, 2411-2418. doi:10.1021/om050111c
5. Lenker, H. K.; Gray, T. G.; Stockland Jr., R. A. *Dalton Trans.* **2012**, *41*, 13274-13276. doi:10.1039/C2DT32129G
6. Kuram, M. R.; Bhanuchandra, M.; Sahoo, A. K. *J. Org. Chem.* **2010**, *75*, 2247-2258. doi:10.1021/jo1000143

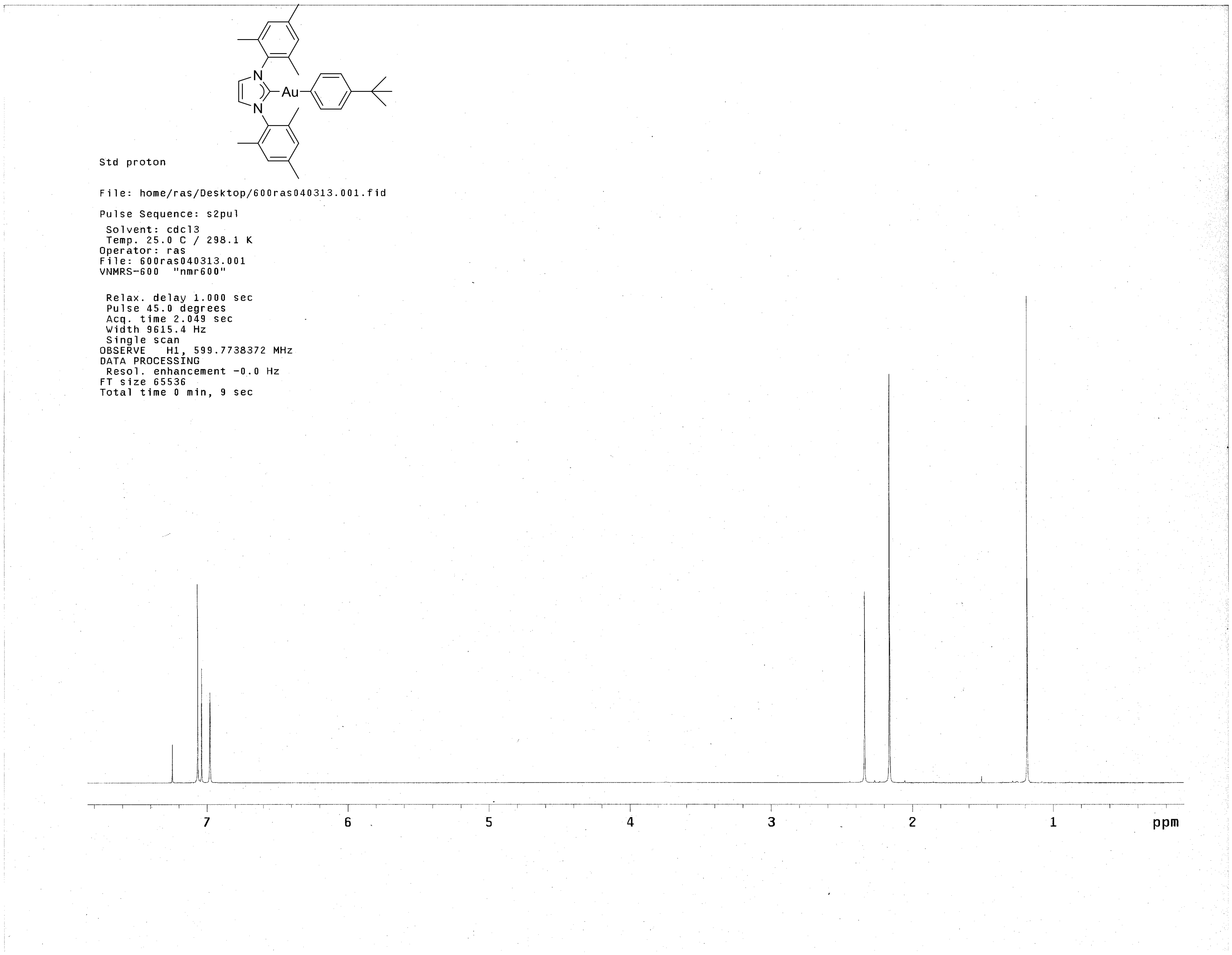

Au
N
N
Std proton
File: home/ras/Desktop/600ras040313.001.fid
Pulse Sequence: s2pul
Solvent: cdcl3
Temp. 25.0 C / 298.1 K
Operator: ras
File: 600ras040313.001
VNMRS-600 "nmr600"
Relax. delay 1.000 sec
Pulse 45.0 degrees
Acq. time 2.049 sec
Width 9615.4 Hz
Single scan
OBSERVE H1, 599.7738372 MHz
DATA PROCESSING
Resol. enhancement -0.0 Hz
FT size 65536
Total time 0 min, 9 sec
7
6
5
4
3
2
1
ppm

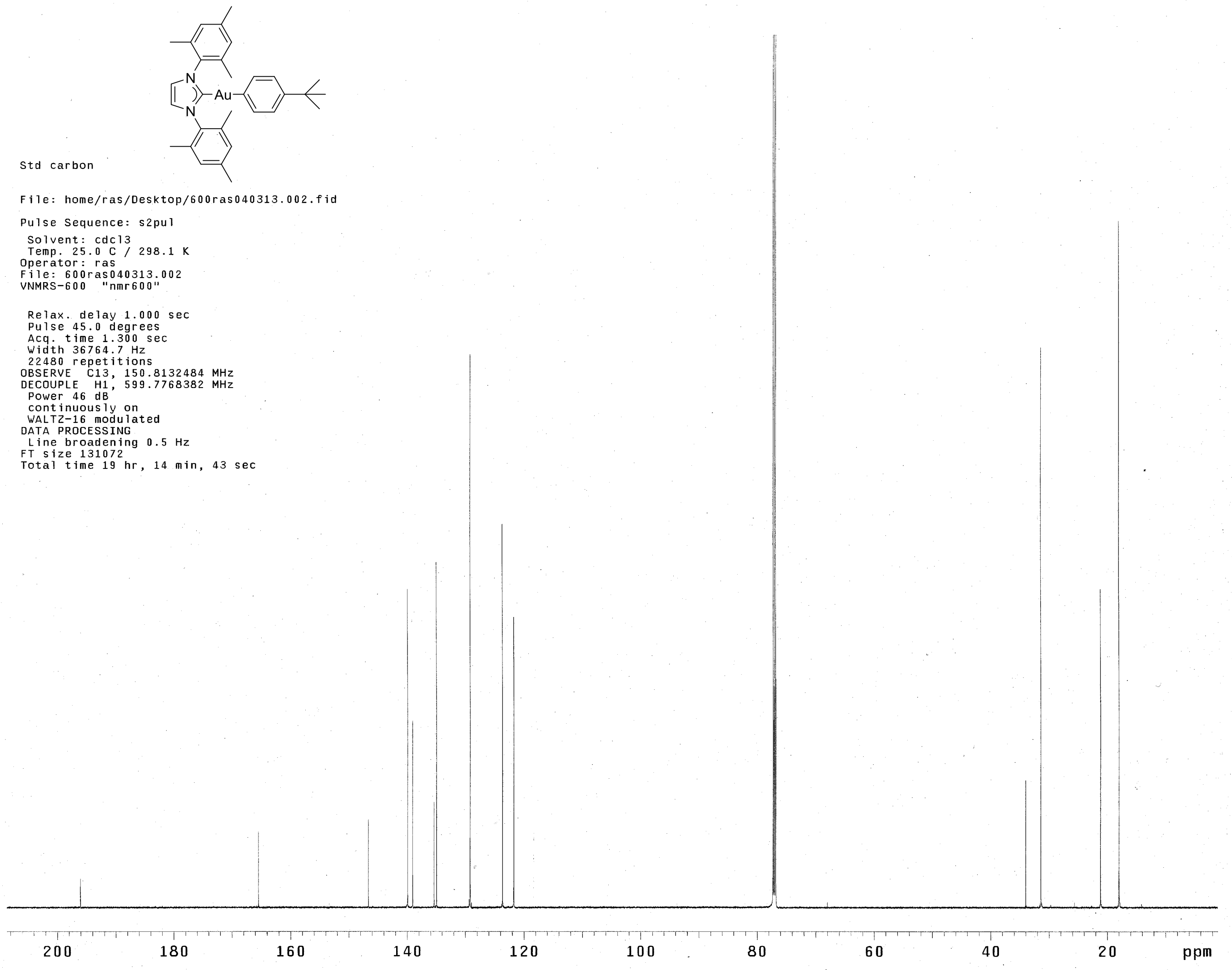

Std carbon

File: home/ras/Desktop/600ras040313.002.fid

Pulse Sequence: s2pul
Solvent: cdcl3
Temp. 25.0 C / 298.1 K
Operator: ras
File: 600ras040313.002
VNMRS-600 "nmr600"

Relax. delay 1.000 sec
Pulse 45.0 degrees
Acq. time 1.300 sec
Width 36764.7 Hz
22480 repetitions
OBSERVE C13, 150.8132484 MHz
DECOUPLE H1, 599.7768382 MHz
Power 46 dB
continuously on
WALTZ-16 modulated
DATA PROCESSING
Line broadening 0.5 Hz
FT size 131072
Total time 19 hr, 14 min, 43 sec

Std proton

Sample Name:

Archive directory:

Sample directory:

FidFile: 600ras032213.006

Pulse Sequence: Proton (s2pul)
Solvent: cdcl3
Data collected on: Mar 27 2013

Temp. 25.0 C / 298.1 K
Operator: ras
VNMRS-600 "nmr600"

Relax. delay 1.000 sec
Pulse 45.0 degrees
Acq. time 2.049 sec
Width 9615.4 Hz
Single scan
OBSERVE H1, 599.7738393 MHz
DATA PROCESSING
Resol. enhancement -0.0 Hz
FT size 65536
Total time 0 min 9 sec

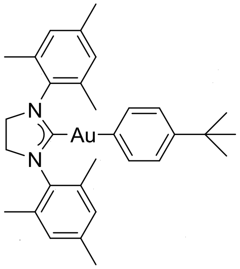

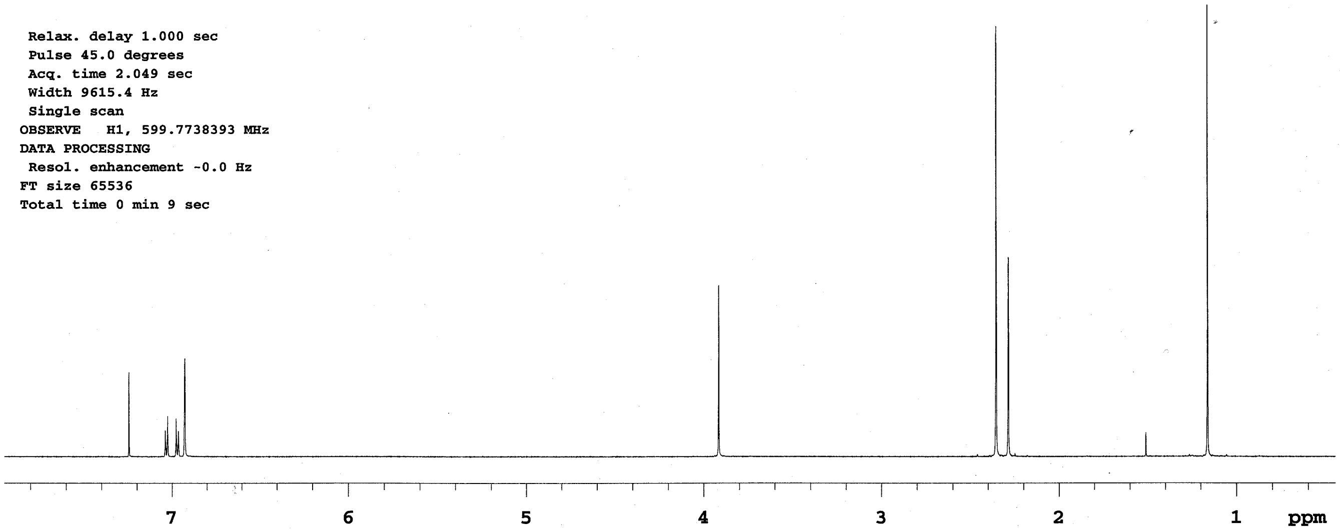

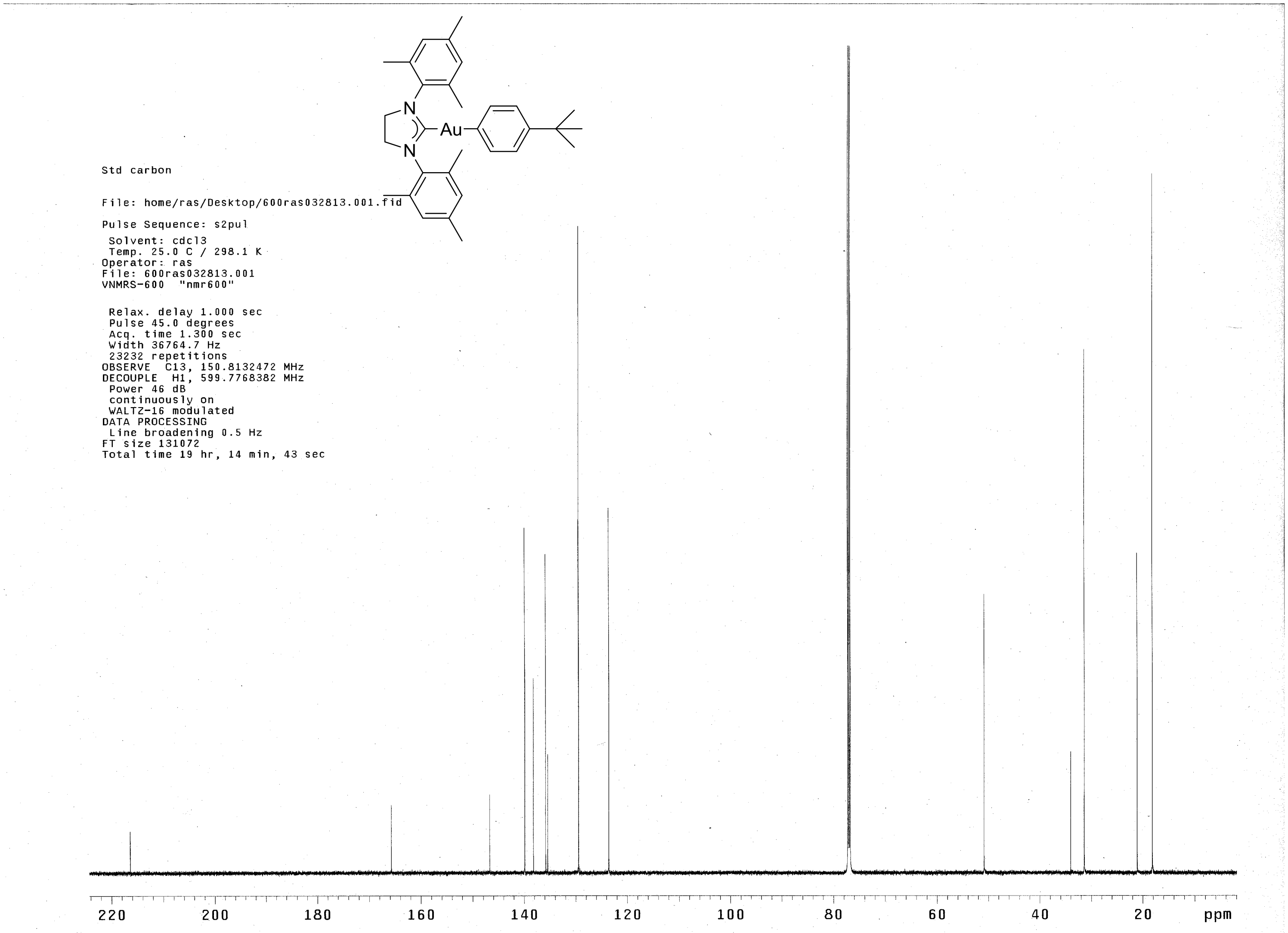
Au
N
N
Std carbon
File: home/ras/Desktop/600ras032813.001.fid
Pulse Sequence: s2pul
Solvent: cdcl3
Temp. 25.0 C / 298.1 K
Operator: ras
File: 600ras032813.001
VNMRS-600 "nmr600"
Relax. delay 1.000 sec
Pulse 45.0 degrees
Acq. time 1.300 sec
Width 36764.7 Hz
23232 repetitions
OBSERVE C13, 150.8132472 MHz
DECOUPLE H1, 599.7768382 MHz
Power 46 dB
continuously on
WALTZ-16 modulated
DATA PROCESSING
Line broadening 0.5 Hz
FT size 131072
Total time 19 hr, 14 min, 43 sec
220 200 180 160 140 120 100 80 60 40 20 ppm

Sample Name:

Data Collected on:
nmr600-vnmrs600
Archive directory:

Sample directory:

FidFile: 600ras052913.004

Pulse Sequence: PROTON (s2pul)
Solvent: cdcl3
Data collected on: May 28 2013

Temp. 25.0 C / 298.1 K
Operator: ras

Relax. delay 1.000 sec
Pulse 45.0 degrees
Acq. time 1.704 sec
Width 9615.4 Hz
Single scan
OBSERVE H1, 599.7738340 MHz
DATA PROCESSING
FT size 32768
Total time 0 min 3 sec

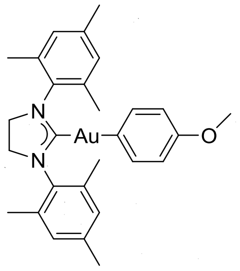

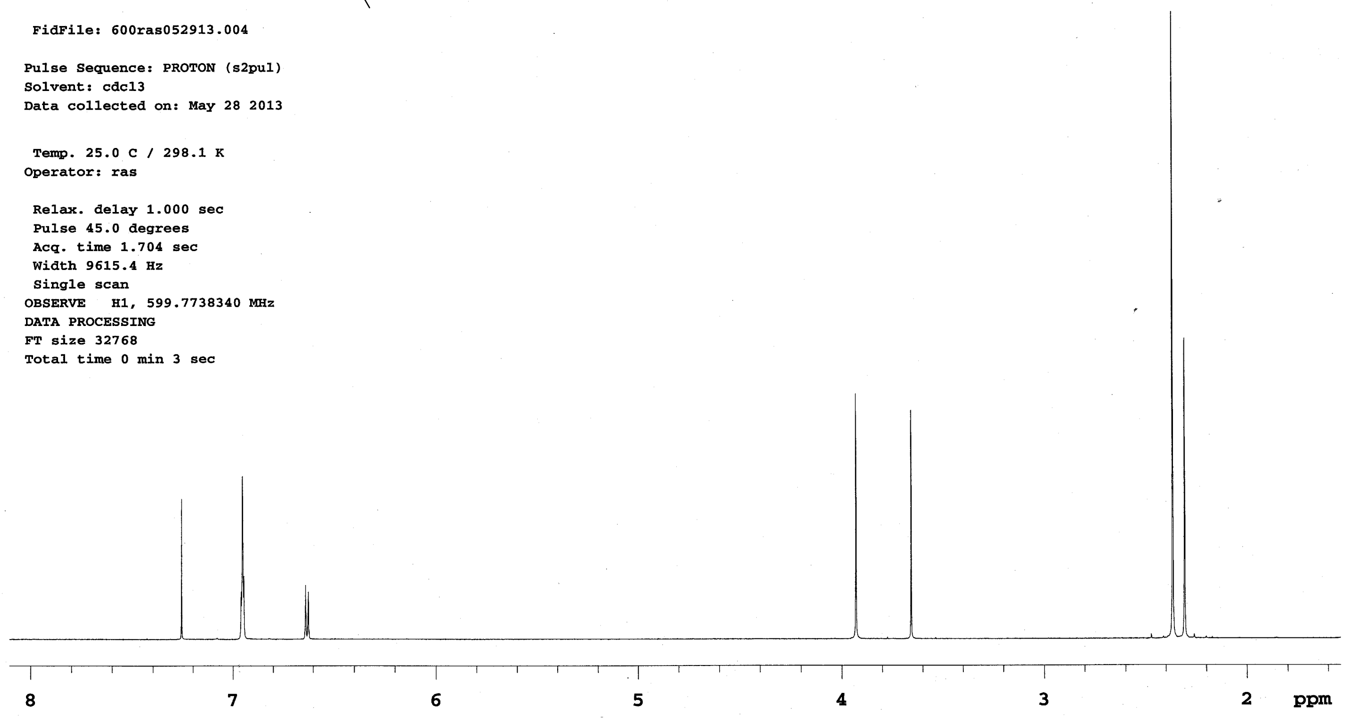

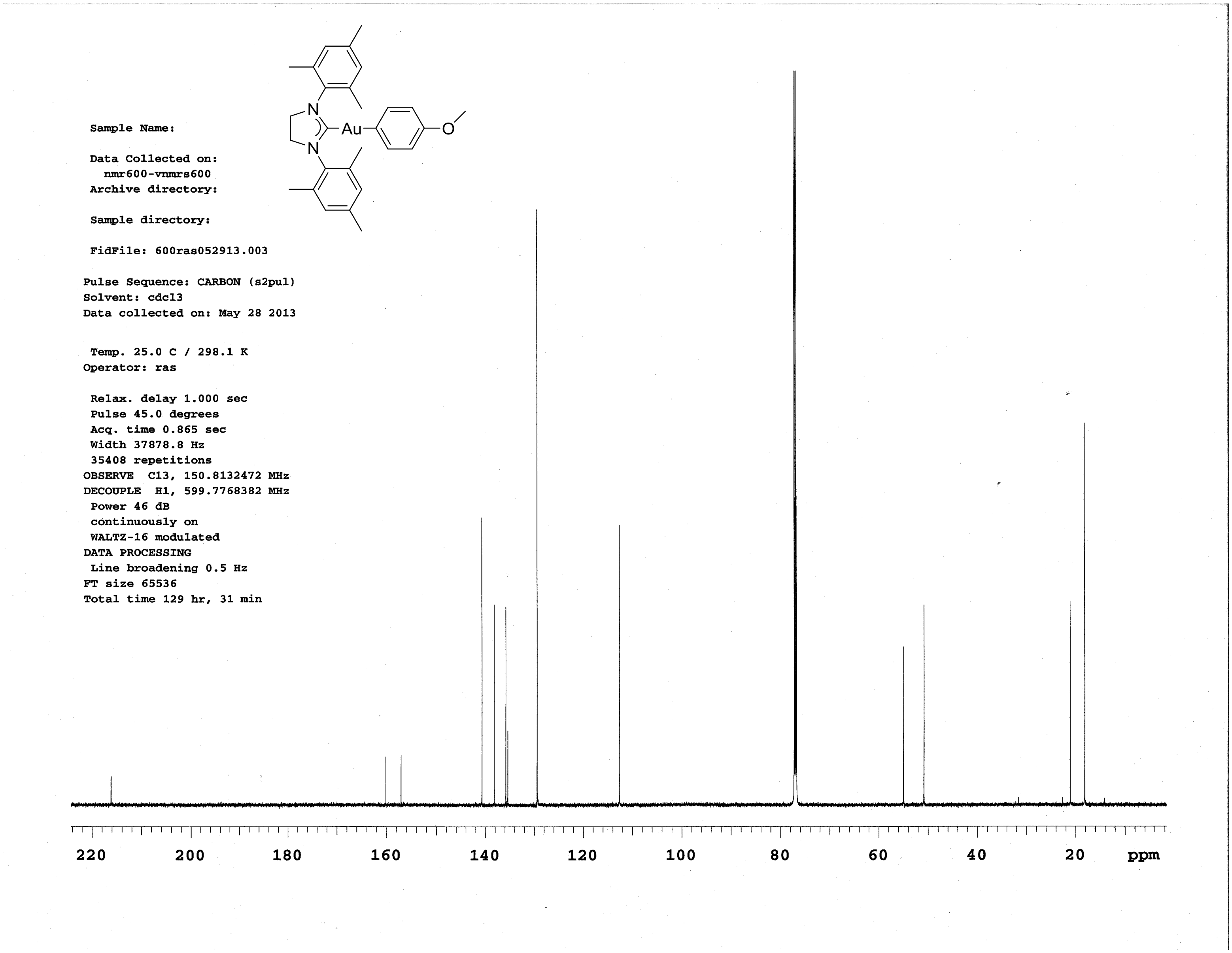
N
N
Au
O
Sample Name:
Data Collected on:
nmr600-vnmrs600
Archive directory:
Sample directory:
FidFile: 600ras052913.003
Pulse Sequence: CARBON (s2pul)
Solvent: cdcl3
Data collected on: May 28 2013
Temp. 25.0 C / 298.1 K
Operator: ras
Relax. delay 1.000 sec
Pulse 45.0 degrees
Acq. time 0.865 sec
Width 37878.8 Hz
35408 repetitions
OBSERVE C13, 150.8132472 MHz
DECOUPLE H1, 599.7768382 MHz
Power 46 dB
continuously on
WALTZ-16 modulated
DATA PROCESSING
Line broadening 0.5 Hz
FT size 65536
Total time 129 hr, 31 min
220
200
180
160
140
120
100
80
60
40
20
ppm

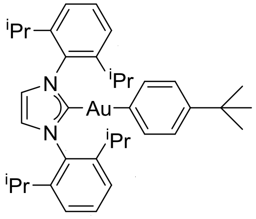

Sample Name:

Data Collected on:
nmr600-vnmrs600
Archive directory:

Sample directory:

FidFile: 600ras061313.007

Pulse Sequence: PROTON (s2pul)
Solvent: cdcl3
Data collected on: Jun 14 2013

Temp. 25.0 C / 298.1 K
Operator: ras

Relax. delay 1.000 sec
Pulse 45.0 degrees
Acq. time 1.704 sec
Width 9615.4 Hz
Single scan
OBSERVE H1, 599.7738340 MHz
DATA PROCESSING
FT size 32768
Total time 0 min 3 sec

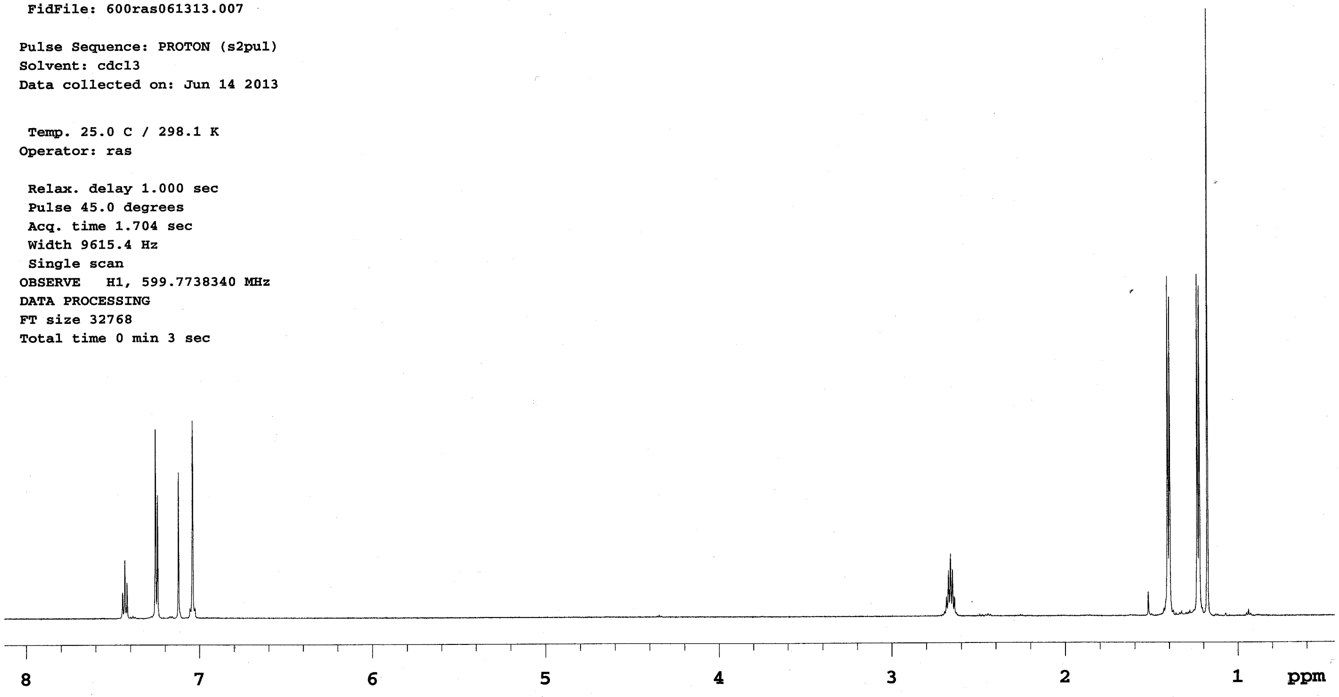

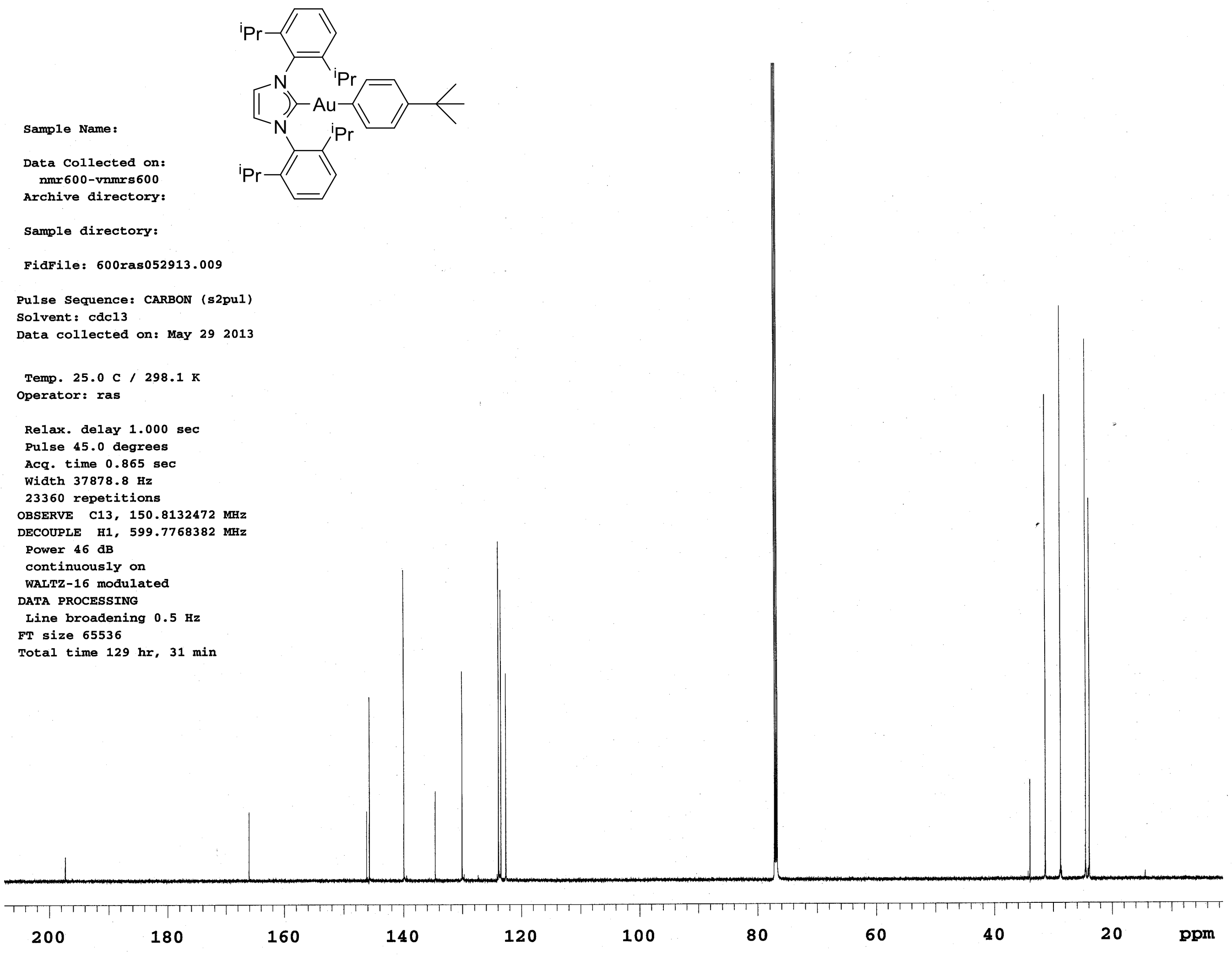

iPr
iPr
N
Au
N
iPr
iPr
Sample Name:
Data Collected on:
nmr600-vnmrs600
Archive directory:
Sample directory:
FidFile: 600ras052913.009
Pulse Sequence: CARBON (s2pul)
Solvent: cdcl3
Data collected on: May 29 2013
Temp. 25.0 C / 298.1 K
Operator: ras
Relax. delay 1.000 sec
Pulse 45.0 degrees
Acq. time 0.865 sec
Width 37878.8 Hz
23360 repetitions
OBSERVE C13, 150.8132472 MHz
DECOUPLE H1, 599.7768382 MHz
Power 46 dB
continuously on
WALTZ-16 modulated
DATA PROCESSING
Line broadening 0.5 Hz
FT size 65536
Total time 129 hr, 31 min
200
180
160
140
120
100
80
60
40
20
ppm

Sample Name:

Data Collected on:
nmr600-vnmrs600
Archive directory:

Sample directory:

FidFile: 600ras061313.008

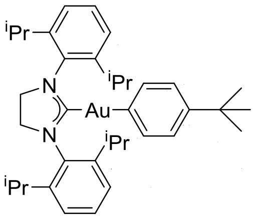

Pulse Sequence: PROTON (s2pul)
Solvent: cdcl3
Data collected on: Jun 14 2013

Temp. 25.0 C / 298.1 K
Operator: ras

Relax. delay 1.000 sec
Pulse 45.0 degrees
Acq. time 1.704 sec
Width 9615.4 Hz
Single scan
OBSERVE H1, 599.7738340 MHz
DATA PROCESSING
FT size 32768
Total time 0 min 3 sec

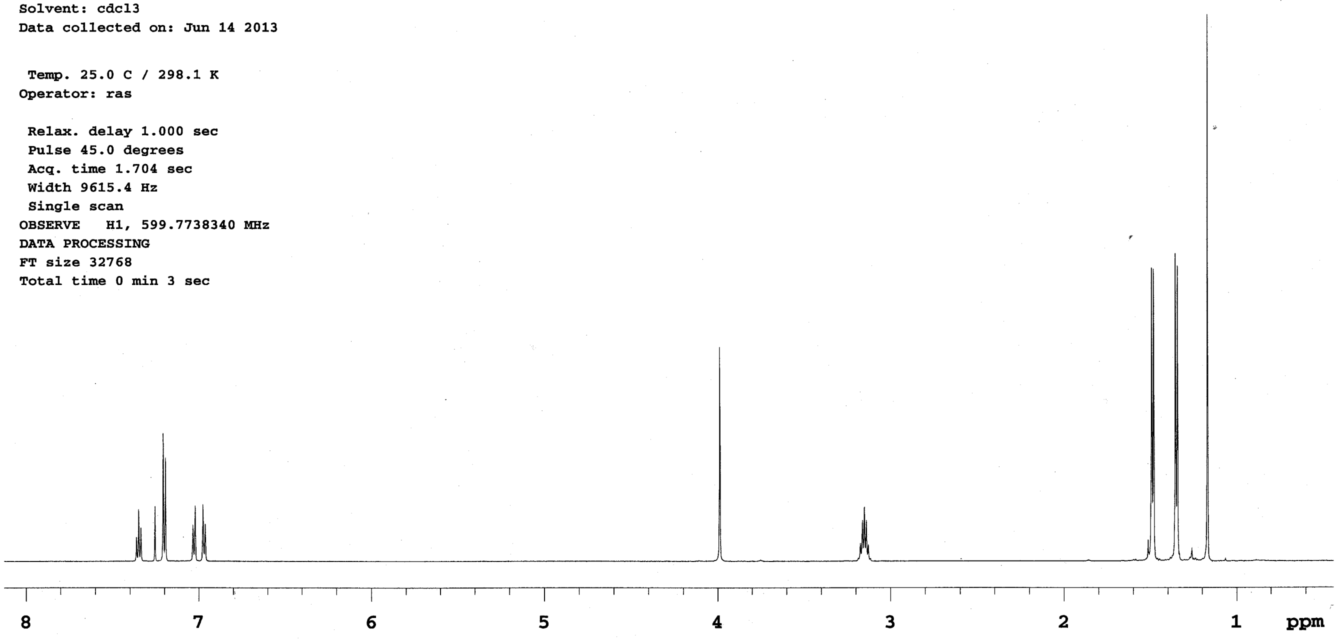

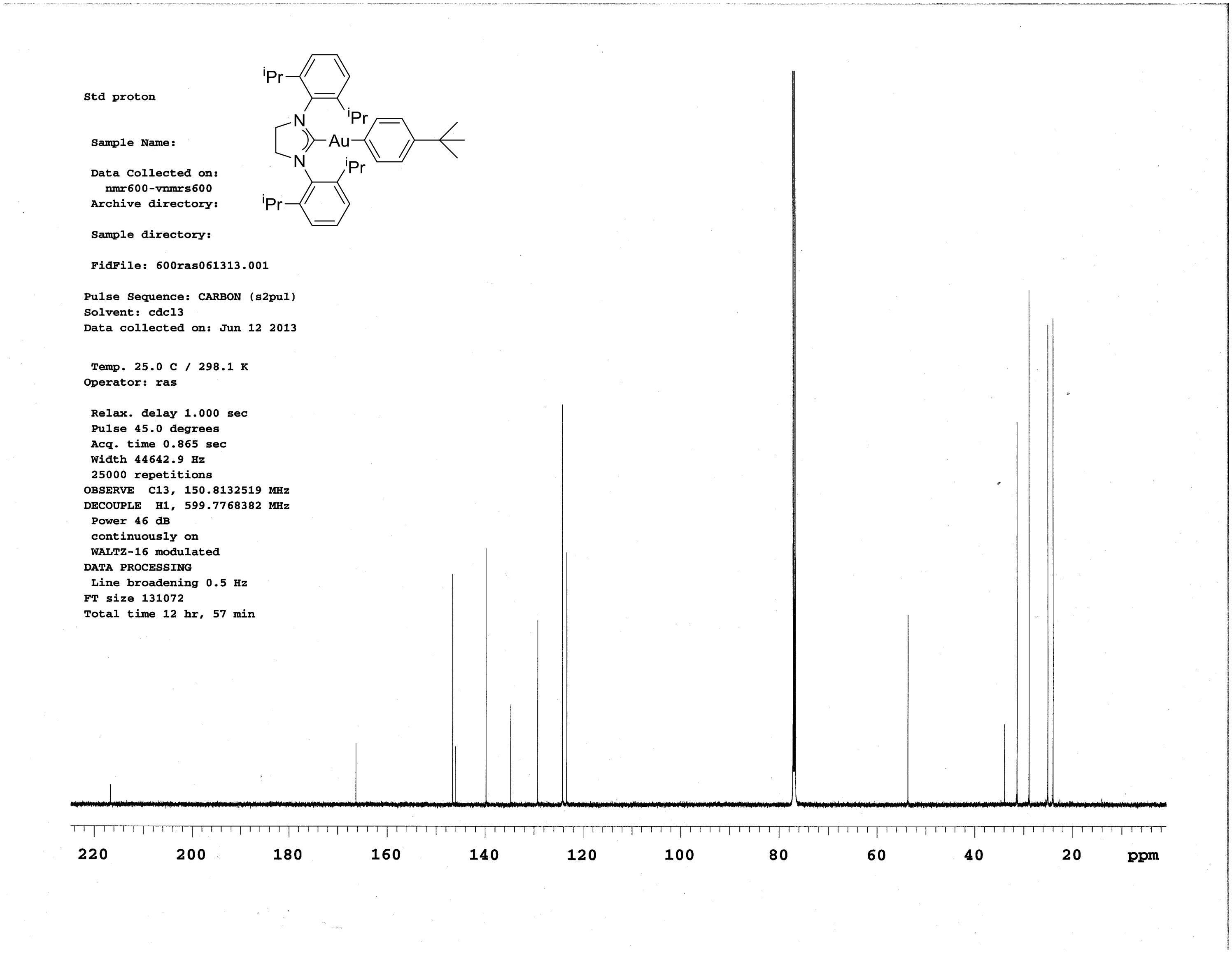
Std proton
Sample Name:
Data Collected on:
nmr600-vnmrs600
Archive directory:
Sample directory:
FidFile: 600ras061313.001
Pulse Sequence: CARBON (s2pul)
Solvent: cdcl3
Data collected on: Jun 12 2013
Temp. 25.0 C / 298.1 K
Operator: ras
Relax. delay 1.000 sec
Pulse 45.0 degrees
Acq. time 0.865 sec
Width 44642.9 Hz
25000 repetitions
OBSERVE C13, 150.8132519 MHz
DECOUPLE H1, 599.7768382 MHz
Power 46 dB
continuously on
WALTZ-16 modulated
DATA PROCESSING
Line broadening 0.5 Hz
FT size 131072
Total time 12 hr, 57 min
iPr
iPr
N
Au
N
iPr
iPr
220
200
180
160
140
120
100
80
60
40
20
ppm

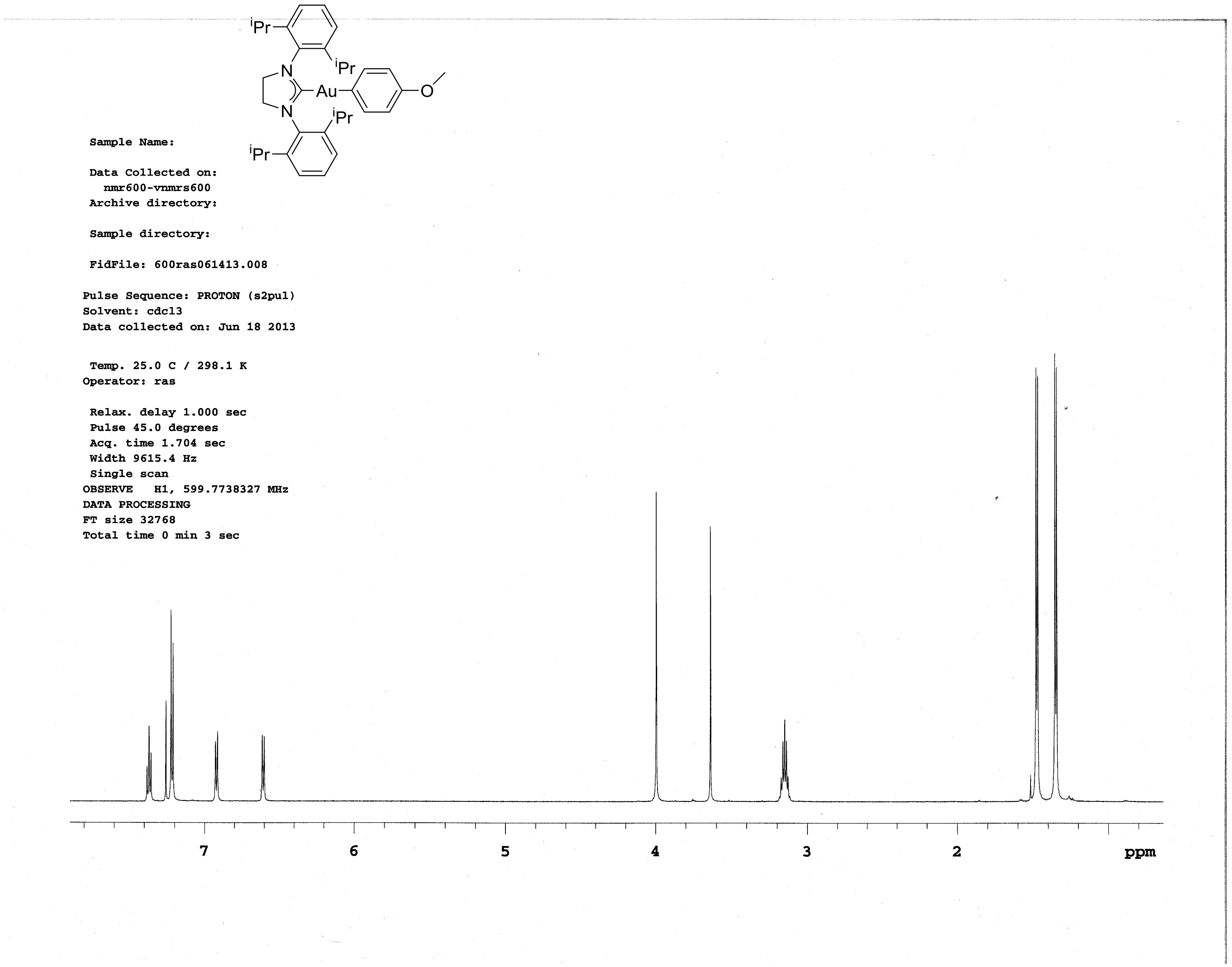

iPr
iPr
N
Au
O
N
iPr
iPr
Sample Name:
Data Collected on:
nmr600-vnmrs600
Archive directory:
Sample directory:
FidFile: 600ras061413.008
Pulse Sequence: PROTON (s2pul)
Solvent: cdcl3
Data collected on: Jun 18 2013
Temp. 25.0 C / 298.1 K
Operator: ras
Relax. delay 1.000 sec
Pulse 45.0 degrees
Acq. time 1.704 sec
Width 9615.4 Hz
Single scan
OBSERVE H1, 599.7738327 MHz
DATA PROCESSING
FT size 32768
Total time 0 min 3 sec
7
6
5
4
3
2
ppm

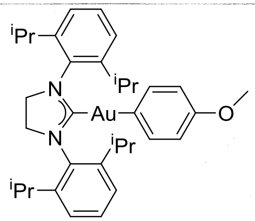

Sample Name:

Data Collected on:
nmr600-vnmrs600
Archive directory:

Sample directory:

FidFile: 600ras061913.001

Pulse Sequence: CARBON (s2pul)
Solvent: cdcl3
Data collected on: Jun 18 2013

Temp. 25.0 C / 298.1 K
Operator: ras

Relax. delay 1.000 sec
Pulse 45.0 degrees
Acq. time 0.865 sec
Width 37878.8 Hz
28576 repetitions
OBSERVE C13, 150.8132478 MHz
DECOUPLE H1, 599.7768382 MHz
Power 46 dB
continuously on
WALTZ-16 modulated
DATA PROCESSING
Line broadening 0.5 Hz
FT size 65536
Total time 129 hr, 31 min

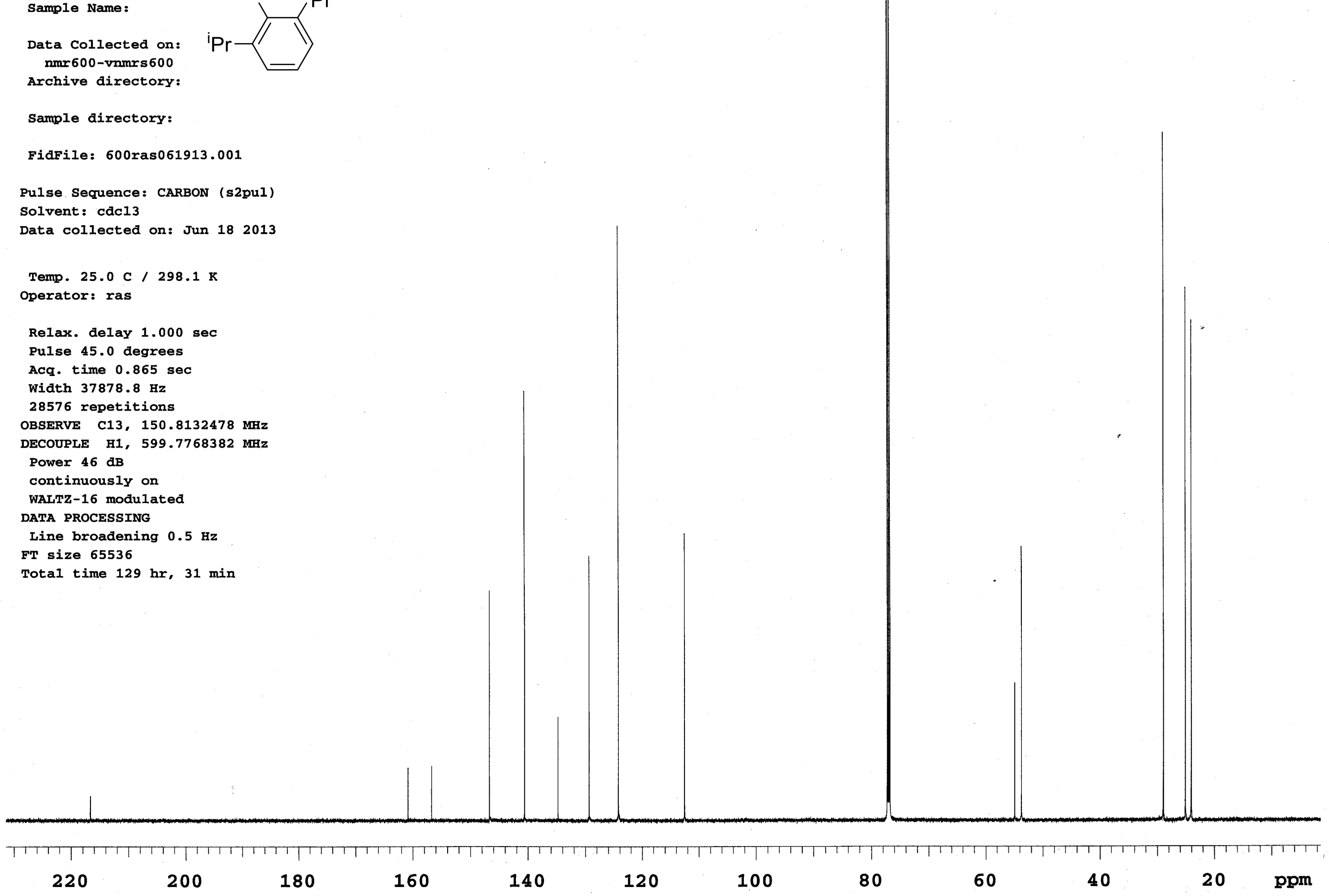

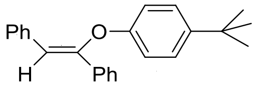

Sample Name:

Data Collected on:
nmr600-vnmrs600
Archive directory:

Sample directory:

FidFile: 600ras062613.006

Pulse Sequence: PROTON (s2pul)
Solvent: cdcl3
Data collected on: Jun 27 2013

Temp. 25.0 C / 298.1 K
Operator: ras

Relax. delay 1.000 sec
Pulse 45.0 degrees
Acq. time 1.704 sec
Width 9615.4 Hz
Single scan
OBSERVE H1, 599.7738483 MHz
DATA PROCESSING
FT size 32768
Total time 0 min 3 sec

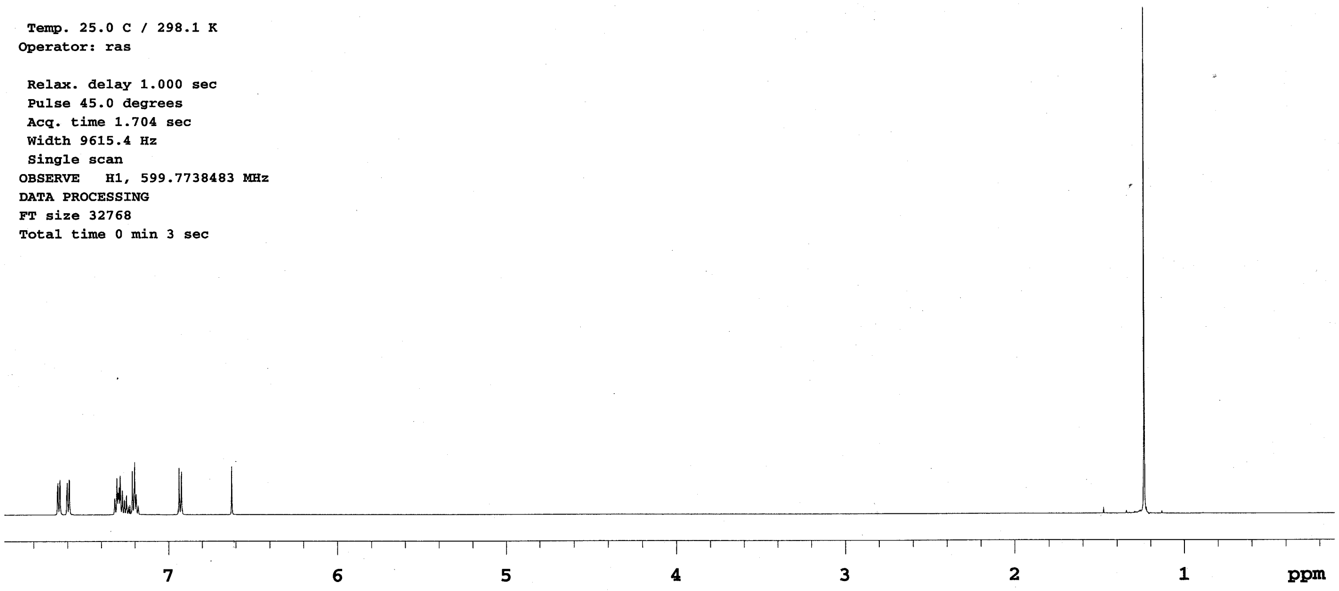

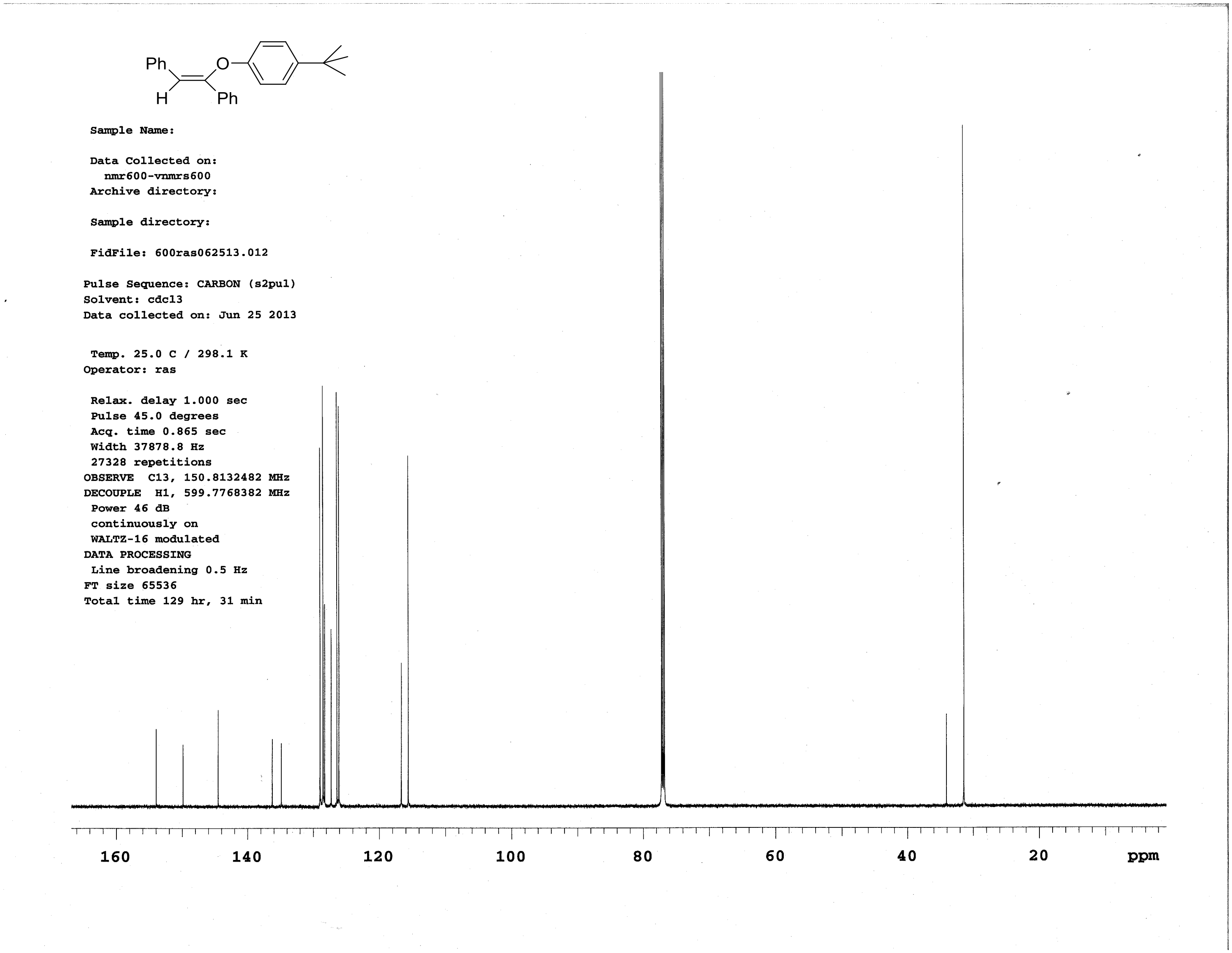
Ph
O
H
Ph
Sample Name:
Data Collected on:
nmr600-vnmrs600
Archive directory:
Sample directory:
FidFile: 600ras062513.012
Pulse Sequence: CARBON (s2pul)
Solvent: cdcl3
Data collected on: Jun 25 2013
Temp. 25.0 C / 298.1 K
Operator: ras
Relax. delay 1.000 sec
Pulse 45.0 degrees
Acq. time 0.865 sec
Width 37878.8 Hz
27328 repetitions
OBSERVE C13, 150.8132482 MHz
DECOUPLE H1, 599.7768382 MHz
Power 46 dB
continuously on
WALTZ-16 modulated
DATA PROCESSING
Line broadening 0.5 Hz
FT size 65536
Total time 129 hr, 31 min
160
140
120
100
80
60
40
20
ppm

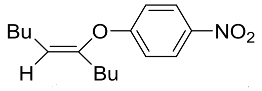

Sample Name:

Data Collected on:
nmr600-vnmrs600
Archive directory:

Sample directory:

FidFile: 600ras062613.011

Pulse Sequence: PROTON (s2pul)
Solvent: cdcl3
Data collected on: Jun 27 2013

Temp. 25.0 C / 298.1 K
Operator: ras

Relax. delay 1.000 sec
Pulse 45.0 degrees
Acq. time 1.704 sec
Width 9615.4 Hz
Single scan
OBSERVE H1, 599.7738423 MHz
DATA PROCESSING
FT size 32768
Total time 0 min 3 sec

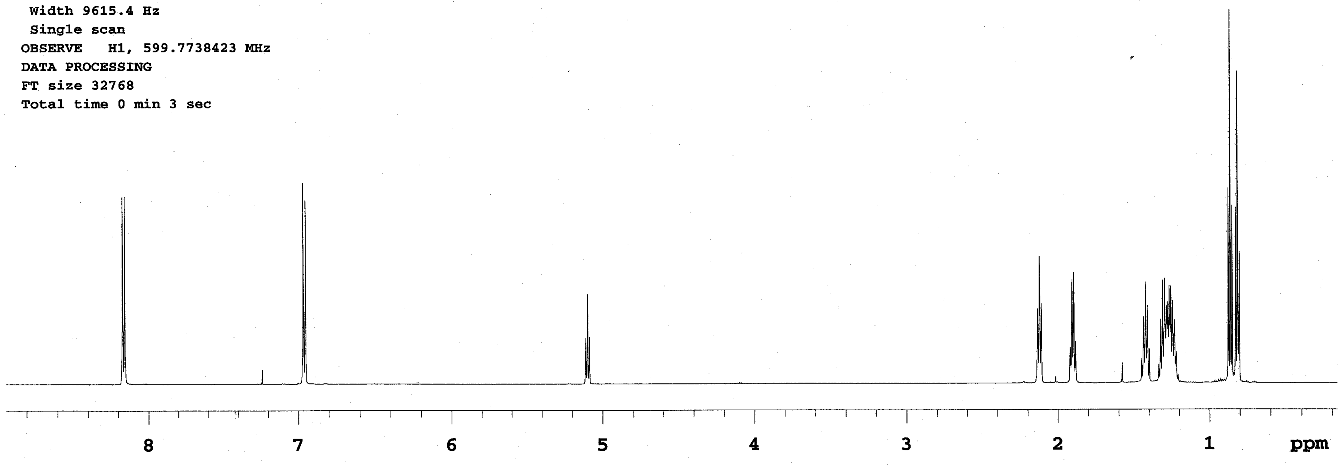

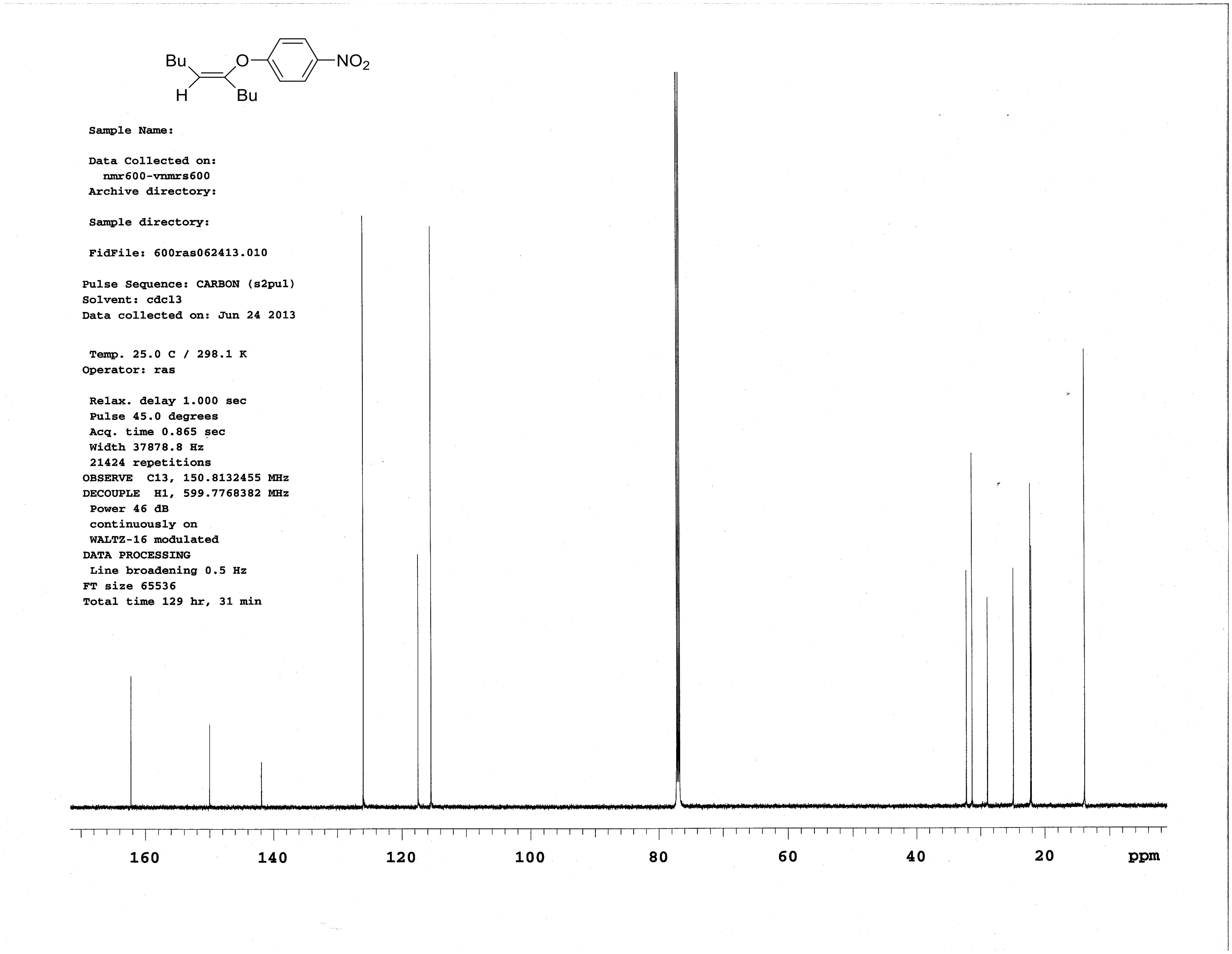
Bu
O
NO2
H
Bu
Sample Name:
Data Collected on:
nmr600-vnmrs600
Archive directory:
Sample directory:
FidFile: 600ras062413.010
Pulse Sequence: CARBON (s2pul)
Solvent: cdcl3
Data collected on: Jun 24 2013
Temp. 25.0 C / 298.1 K
Operator: ras
Relax. delay 1.000 sec
Pulse 45.0 degrees
Acq. time 0.865 sec
Width 37878.8 Hz
21424 repetitions
OBSERVE C13, 150.8132455 MHz
DECOUPLE H1, 599.7768382 MHz
Power 46 dB
continuously on
WALTZ-16 modulated
DATA PROCESSING
Line broadening 0.5 Hz
FT size 65536
Total time 129 hr, 31 min
160
140
120
100
80
60
40
20
ppm

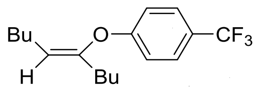

Sample Name:

Data Collected on:
nmr600-vnmrs600
Archive directory:

Sample directory:

FidFile: 600ras062513.008

Pulse Sequence: PROTON (s2pul)
Solvent: cdcl3
Data collected on: Jun 25 2013

Temp. 25.0 C / 298.1 K
Operator: ras

Relax. delay 1.000 sec
Pulse 45.0 degrees
Acq. time 1.704 sec
Width 9615.4 Hz
Single scan
OBSERVE H1, 599.7738393 MHz
DATA PROCESSING
FT size 32768
Total time 0 min 3 sec

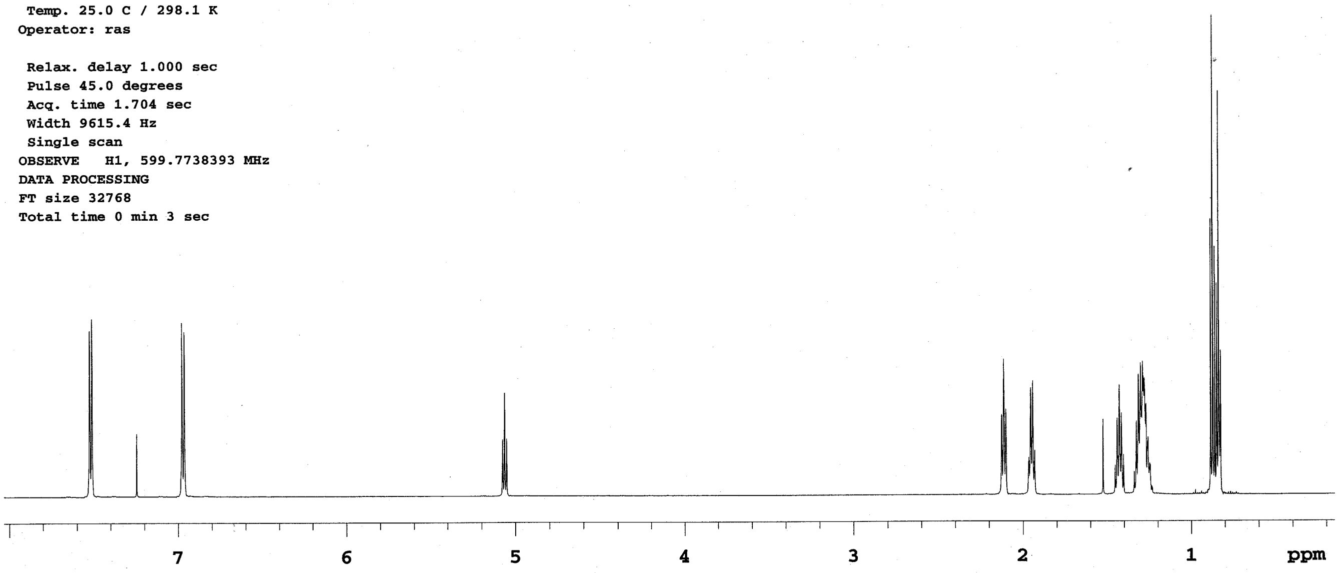

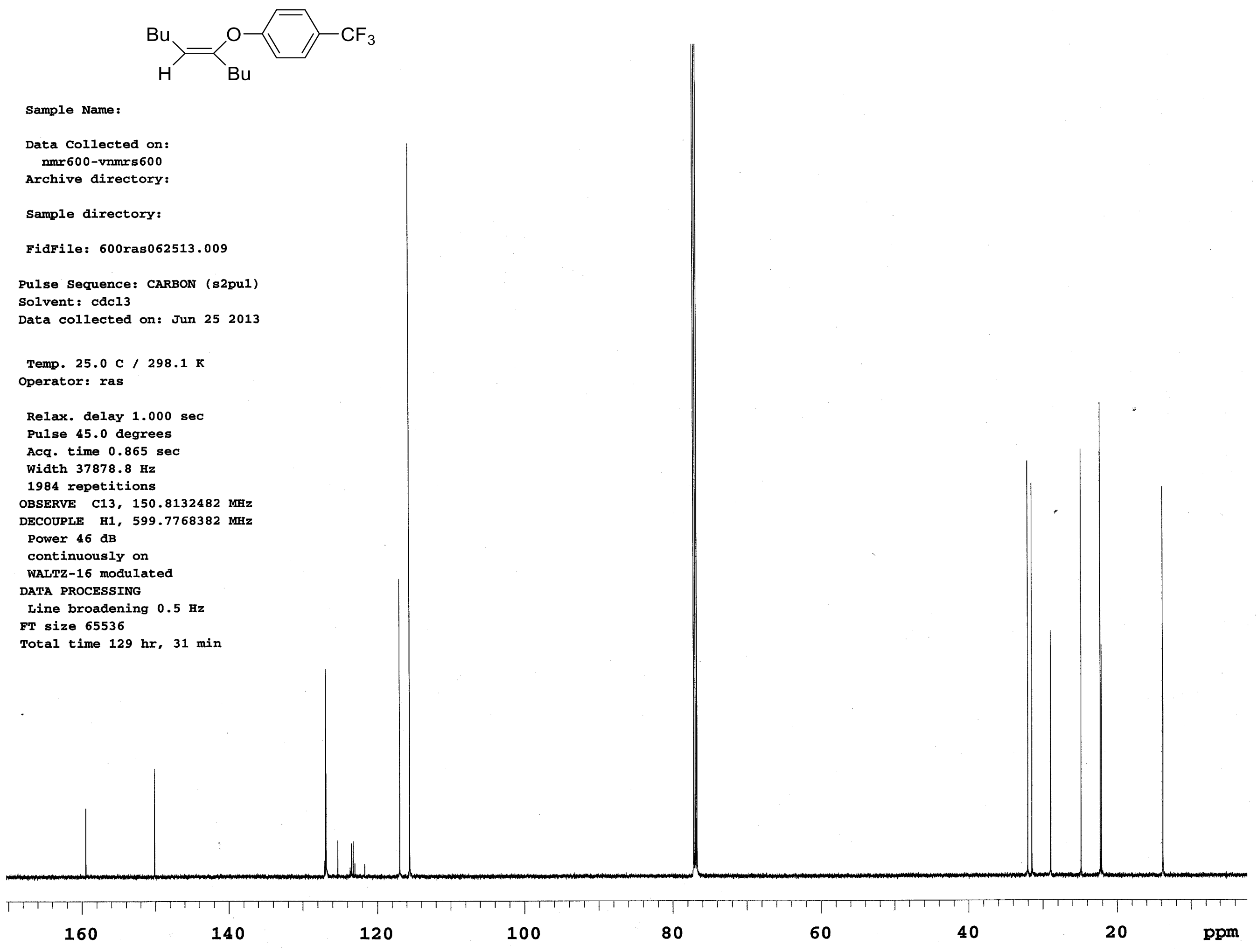

Sample Name:

Data Collected on:
nmr600-vnmrs600
Archive directory:

Sample directory:

FidFile: 600ras062513.009

Pulse Sequence: CARBON (s2pul)
Solvent: cdcl3
Data collected on: Jun 25 2013

Temp. 25.0 C / 298.1 K
Operator: ras

Relax. delay 1.000 sec
Pulse 45.0 degrees
Acq. time 0.865 sec
Width 37878.8 Hz
1984 repetitions
OBSERVE C13, 150.8132482 MHz
DECOUPLE H1, 599.7768382 MHz
Power 46 dB
continuously on
WALTZ-16 modulated
DATA PROCESSING
Line broadening 0.5 Hz
FT size 65536
Total time 129 hr, 31 min

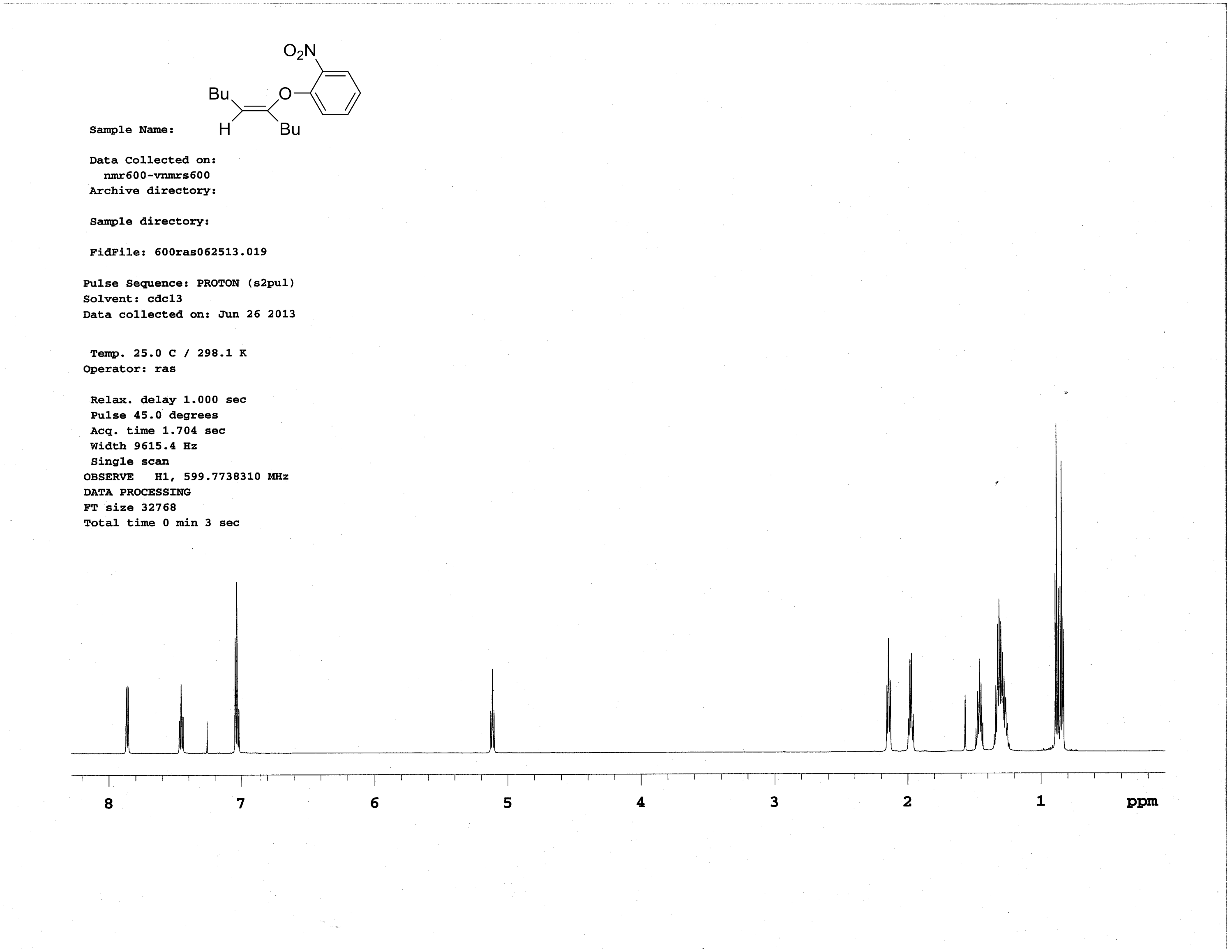
O2N
Bu
O
H
Bu
Sample Name:
Data Collected on:
nmr600-vnmrs600
Archive directory:
Sample directory:
FidFile: 600ras062513.019
Pulse Sequence: PROTON (s2pul)
Solvent: cdcl3
Data collected on: Jun 26 2013
Temp. 25.0 C / 298.1 K
Operator: ras
Relax. delay 1.000 sec
Pulse 45.0 degrees
Acq. time 1.704 sec
Width 9615.4 Hz
Single scan
OBSERVE H1, 599.7738310 MHz
DATA PROCESSING
FT size 32768
Total time 0 min 3 sec
8
7
6
5
4
3
2
1
ppm

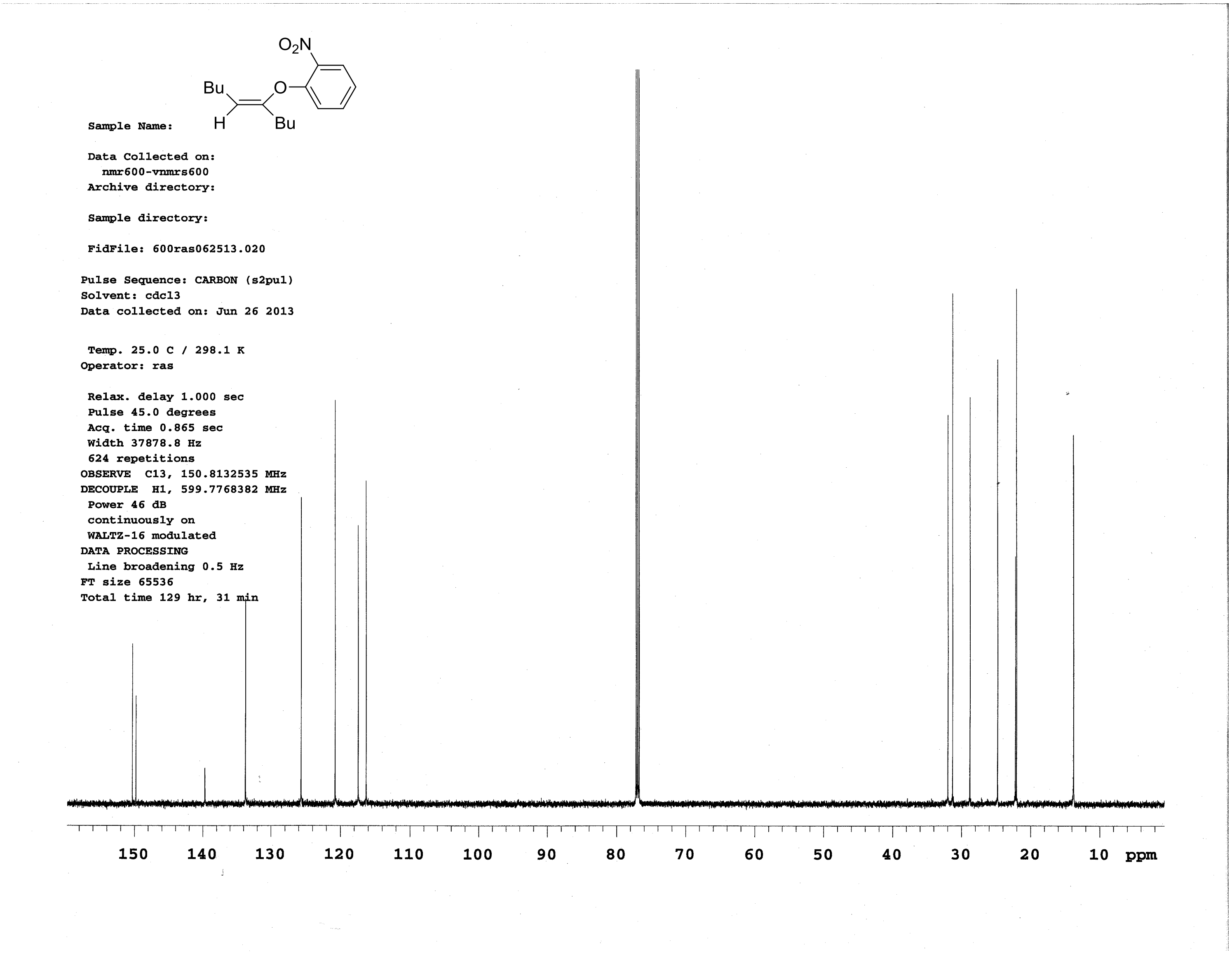
O_2N
Bu
O
H
Bu
Sample Name:
Data Collected on:
nmr600-vnmrs600
Archive directory:
Sample directory:
FidFile: 600ras062513.020
Pulse Sequence: CARBON (s2pul)
Solvent: cdcl3
Data collected on: Jun 26 2013
Temp. 25.0 C / 298.1 K
Operator: ras
Relax. delay 1.000 sec
Pulse 45.0 degrees
Acq. time 0.865 sec
Width 37878.8 Hz
624 repetitions
OBSERVE C13, 150.8132535 MHz
DECOUPLE H1, 599.7768382 MHz
Power 46 dB
continuously on
WALTZ-16 modulated
DATA PROCESSING
Line broadening 0.5 Hz
FT size 65536
Total time 129 hr, 31 min
150 140 130 120 110 100 90 80 70 60 50 40 30 20 10 ppm

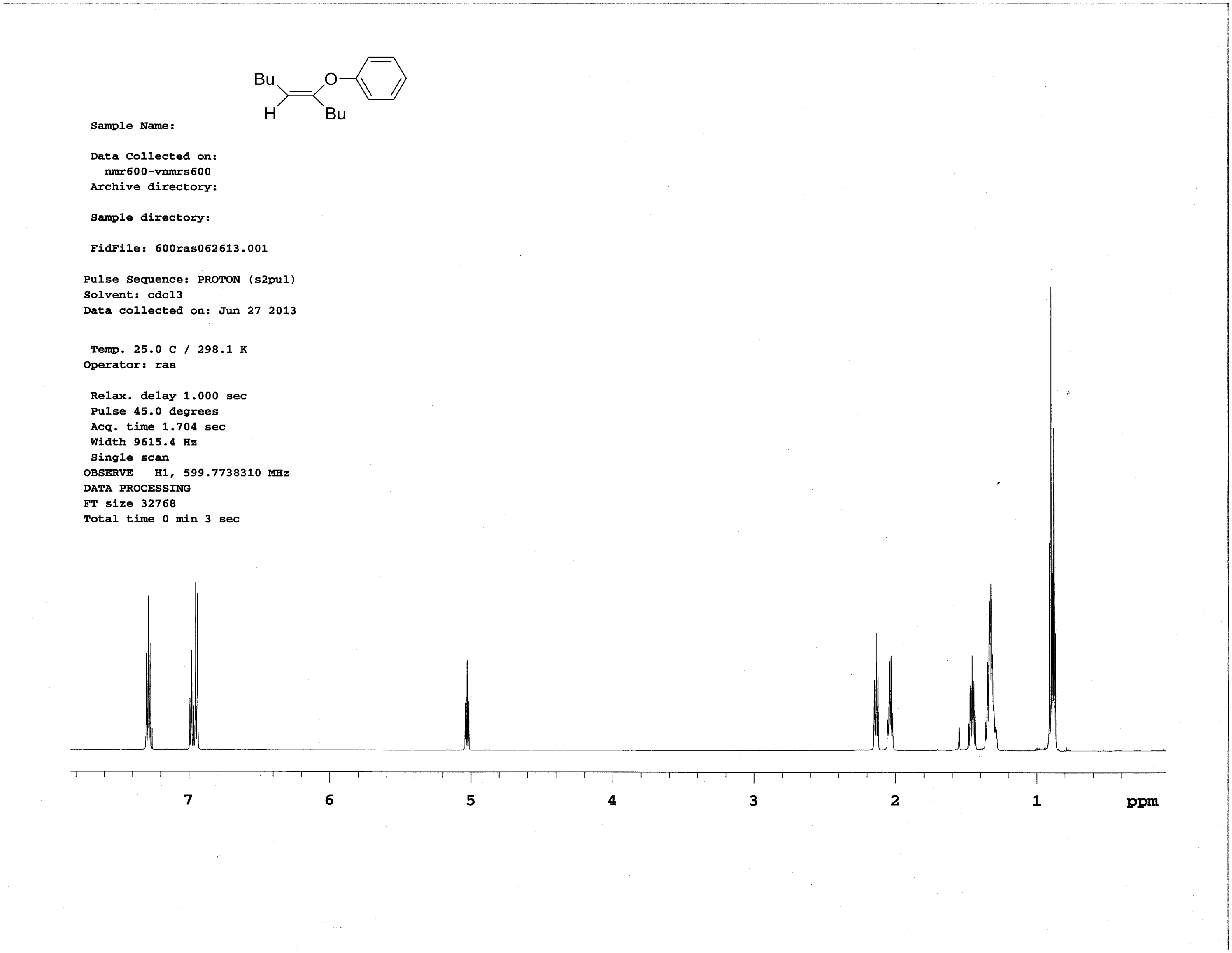

Bu
O
H
Bu
Sample Name:
Data Collected on:
nmr600-vnmrs600
Archive directory:
Sample directory:
FidFile: 600ras062613.001
Pulse Sequence: PROTON (s2pul)
Solvent: cdcl3
Data collected on: Jun 27 2013
Temp. 25.0 C / 298.1 K
Operator: ras
Relax. delay 1.000 sec
Pulse 45.0 degrees
Acq. time 1.704 sec
Width 9615.4 Hz
Single scan
OBSERVE H1, 599.7738310 MHz
DATA PROCESSING
FT size 32768
Total time 0 min 3 sec
7
6
5
4
3
2
1
ppm

Sample Name:

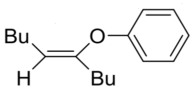

Data Collected on:
nmr600-vnmrs600
Archive directory:

Sample directory:

FidFile: 600ras062613.002

Pulse Sequence: CARBON (s2pul)
Solvent: cdcl3
Data collected on: Jun 27 2013

Temp. 25.0 C / 298.1 K
Operator: ras

Relax. delay 1.000 sec
Pulse 45.0 degrees
Acq. time 0.865 sec
Width 37878.8 Hz
64 repetitions
OBSERVE C13, 150.8132526 MHz
DECOUPLE H1, 599.7768382 MHz
Power 46 dB
continuously on
WALTZ-16 modulated
DATA PROCESSING
Line broadening 0.5 Hz
FT size 65536
Total time 129 hr, 31 min

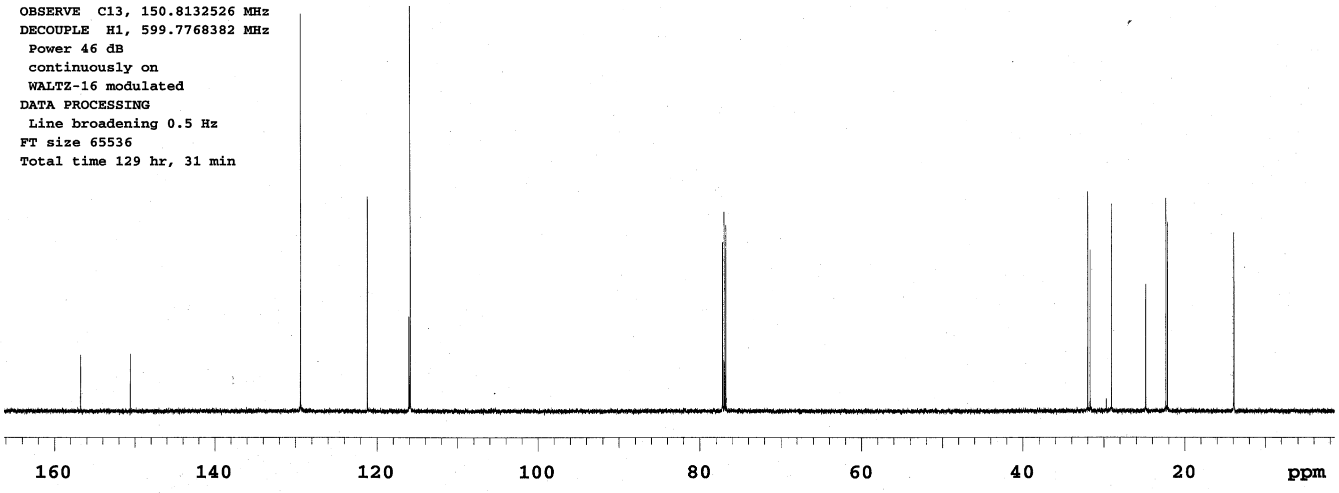

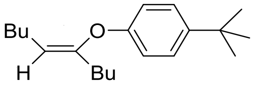

Sample Name:

Data Collected on:
nmr600-vnmrs600
Archive directory:

Sample directory:

FidFile: 600ras062513.004

Pulse Sequence: PROTON (s2pul)
Solvent: cdcl3
Data collected on: Jun 25 2013

Temp. 25.0 C / 298.1 K
Operator: ras

Relax. delay 1.000 sec
Pulse 45.0 degrees
Acq. time 1.704 sec
Width 9615.4 Hz
Single scan
OBSERVE H1, 599.7738393 MHz
DATA PROCESSING
FT size 32768
Total time 0 min 3 sec

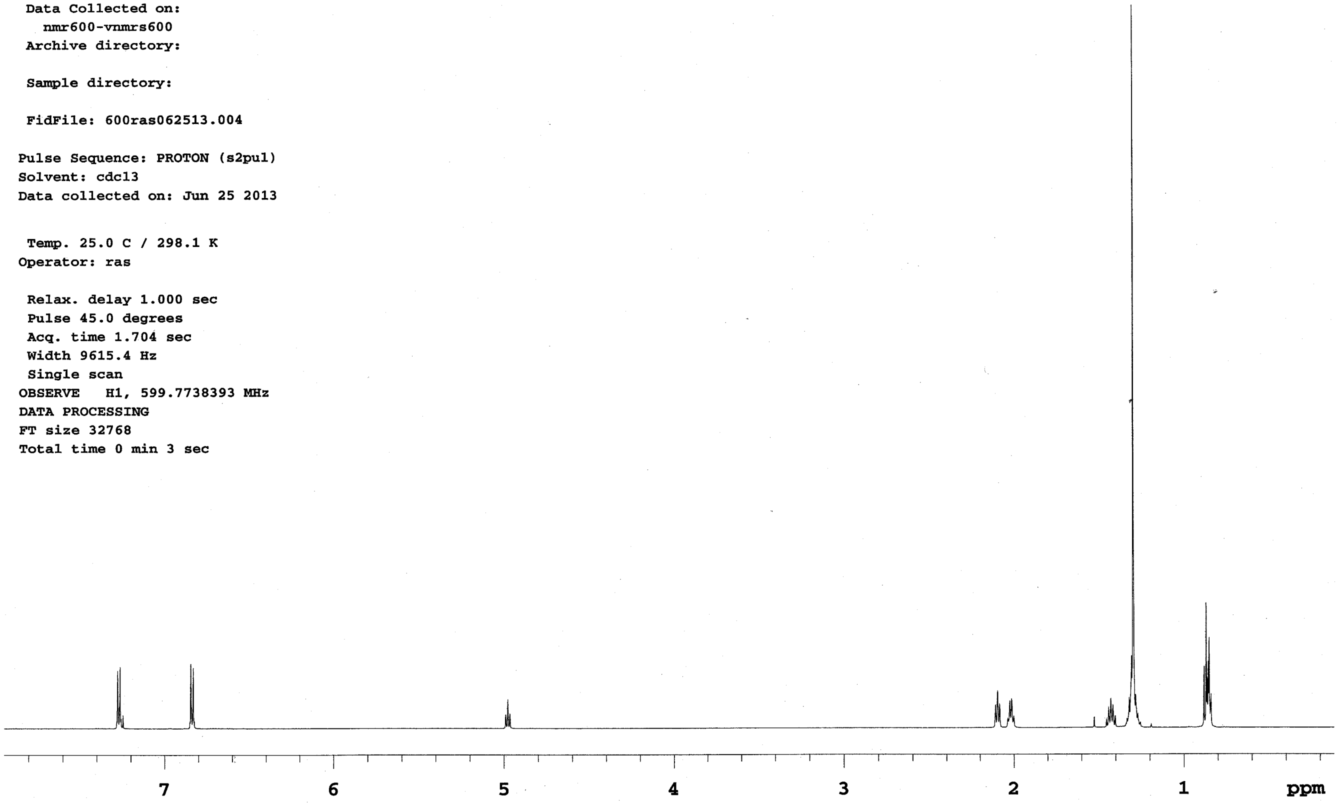

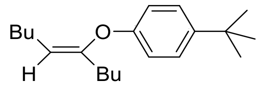

Sample Name:

Data Collected on:
nmr600-vnmrs600
Archive directory:

Sample directory:

FidFile: 600ras062513.005

Pulse Sequence: CARBON (s2pul)
Solvent: cdcl3
Data collected on: Jun 25 2013

Temp. 25.0 C / 298.1 K
Operator: ras

Relax. delay 1.000 sec
Pulse 45.0 degrees
Acq. time 0.865 sec
Width 37878.8 Hz
624 repetitions
OBSERVE C13, 150.8132455 MHz
DECOUPLE H1, 599.7768382 MHz
Power 46 dB
continuously on
WALTZ-16 modulated
DATA PROCESSING
Line broadening 0.5 Hz
FT size 65536
Total time 129 hr, 31 min

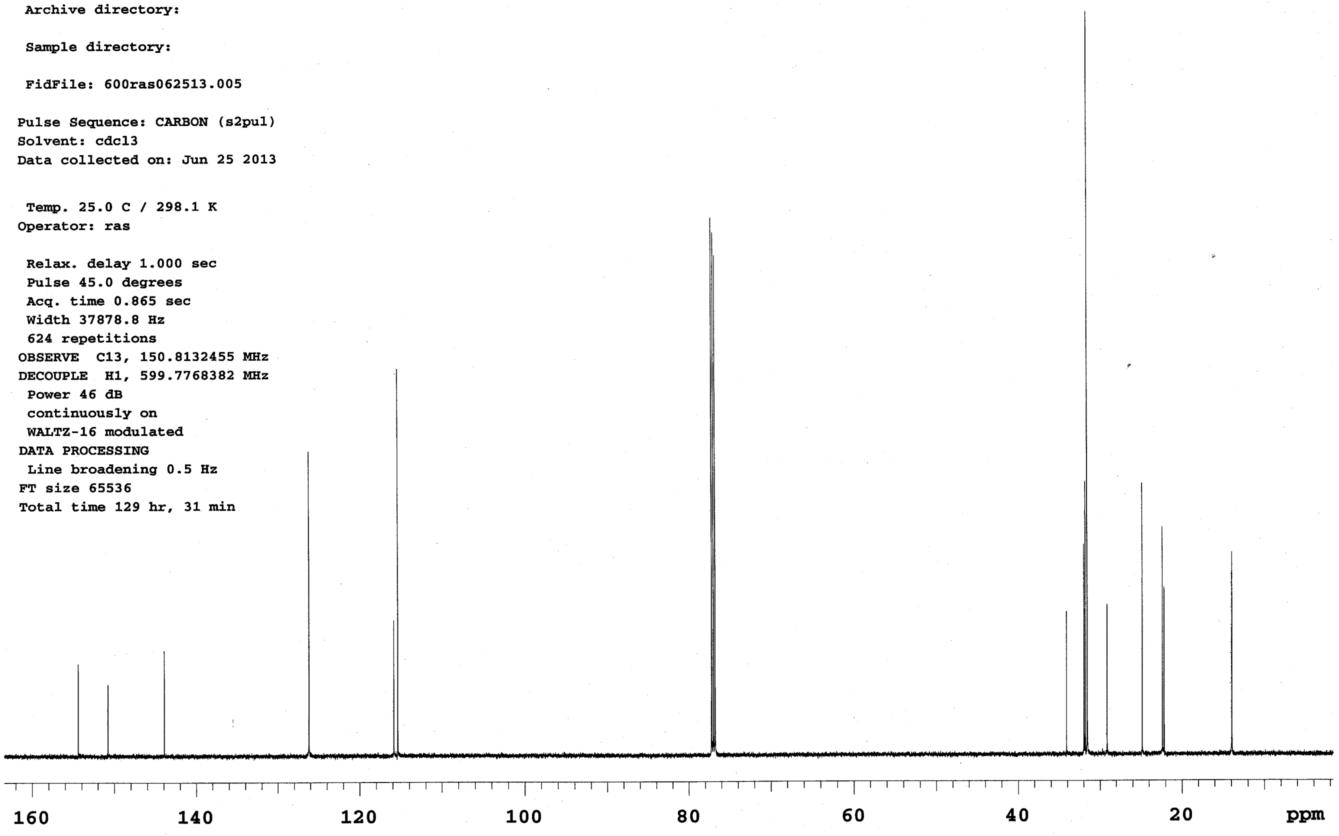

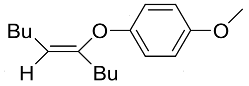

Sample Name:

Data Collected on:
nmr600-vnmrs600
Archive directory:

Sample directory:

FidFile: PROTON

Pulse Sequence: PROTON (s2pul)
Solvent: cdcl3
Data collected on: Jun 27 2013

Temp. 25.0 C / 298.1 K
Operator: ras

Relax. delay 1.000 sec
Pulse 45.0 degrees
Acq. time 1.704 sec
Width 9615.4 Hz
Single scan
OBSERVE H1, 599.7738483 MHz
DATA PROCESSING
FT size 32768
Total time 0 min 3 sec

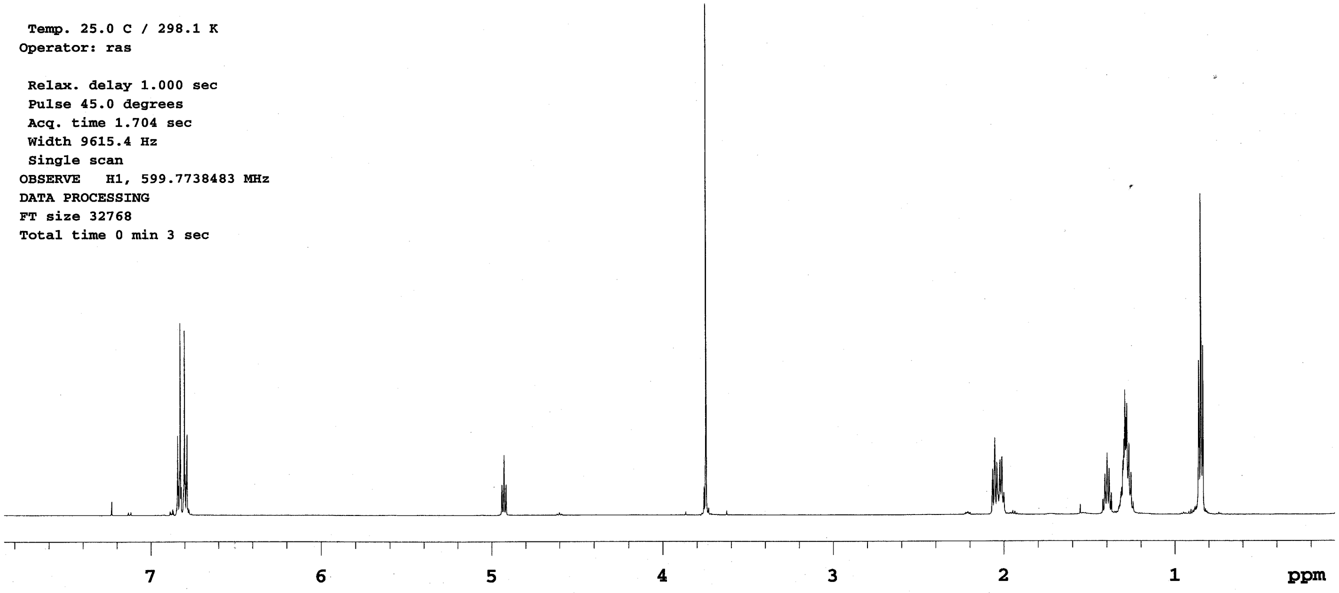

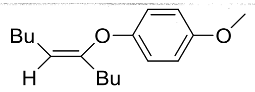

Sample Name:

Data Collected on:
nmr600-vnmrs600
Archive directory:

Sample directory:

FidFile: 600ras062413.002

Pulse Sequence: CARBON (s2pul)
Solvent: cdcl3
Data collected on: Jun 24 2013

Temp. 25.0 C / 298.1 K
Operator: ras

Relax. delay 1.000 sec
Pulse 45.0 degrees
Acq. time 0.865 sec
Width 37878.8 Hz
384 repetitions
OBSERVE C13, 150.8132510 MHz
DECOUPLE H1, 599.7768382 MHz
Power 46 dB
continuously on
WALTZ-16 modulated
DATA PROCESSING
Line broadening 0.5 Hz
FT size 65536
Total time 129 hr, 31 min

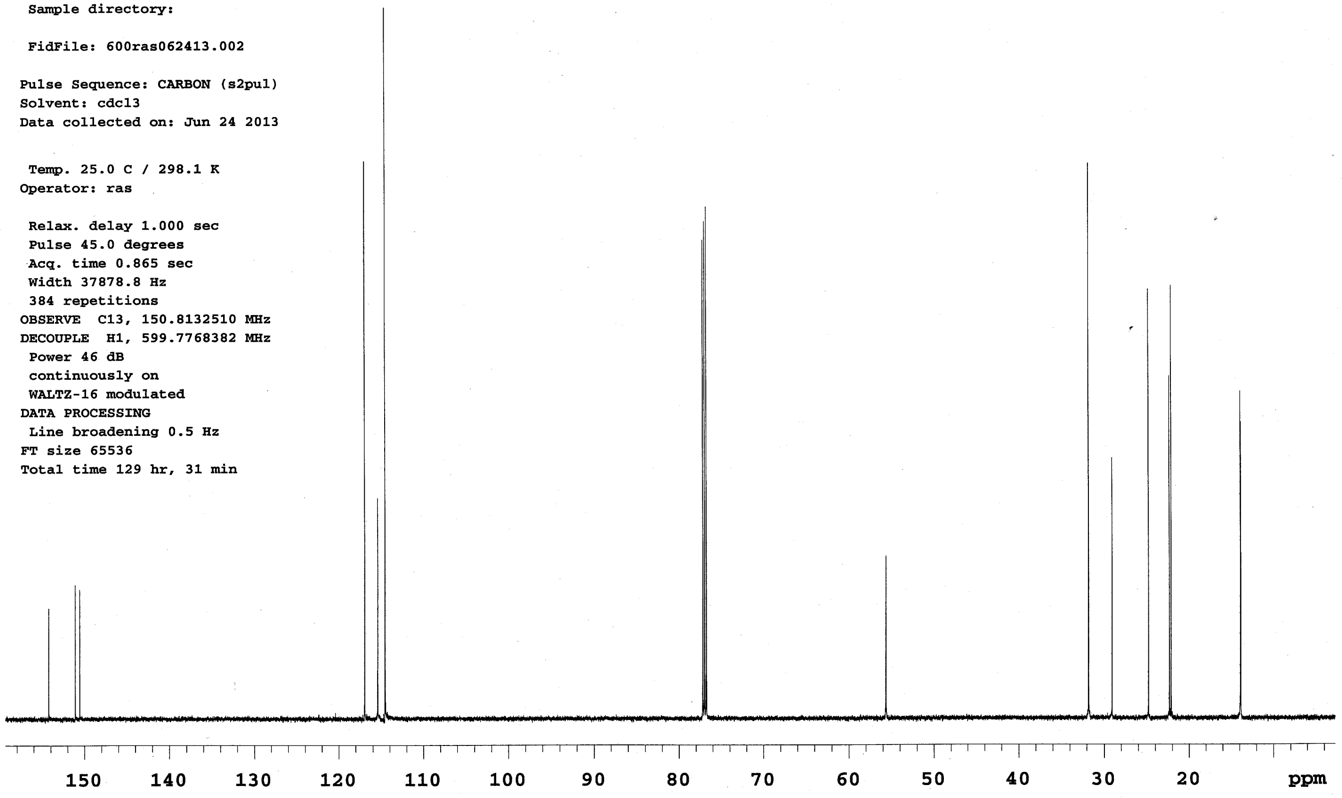

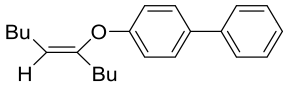
Bu
O
H
Bu

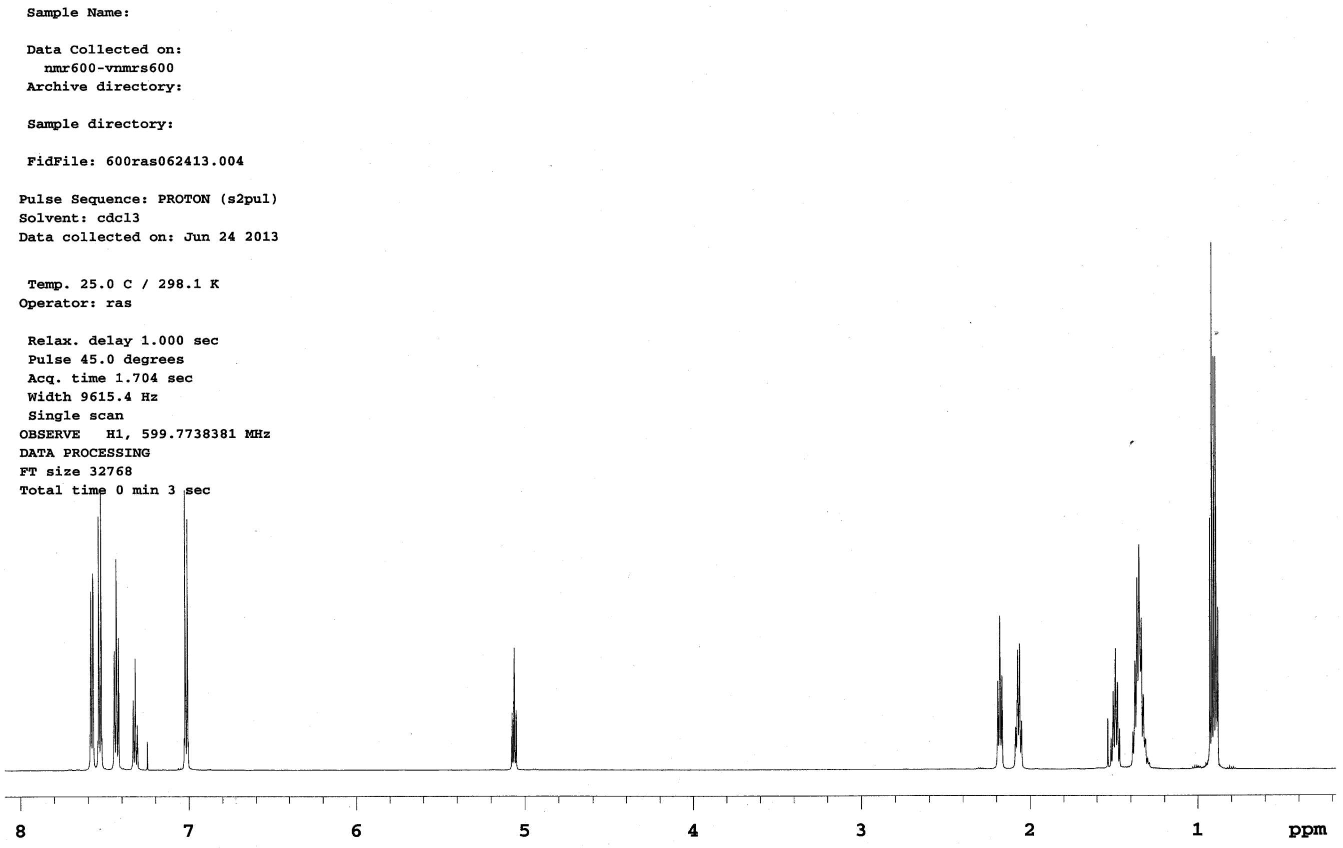
Sample Name:
Data Collected on:
nmr600-vnmrs600
Archive directory:
Sample directory:
FidFile: 600ras062413.004
Pulse Sequence: PROTON (s2pul)
Solvent: cdcl3
Data collected on: Jun 24 2013
Temp. 25.0 C / 298.1 K
Operator: ras
Relax. delay 1.000 sec
Pulse 45.0 degrees
Acq. time 1.704 sec
Width 9615.4 Hz
Single scan
OBSERVE H1, 599.7738381 MHz
DATA PROCESSING
FT size 32768
Total time 0 min 3 sec
8
7
6
5
4
3
2
1
ppm

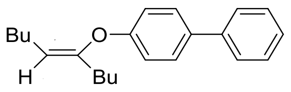

Sample Name:

Data Collected on:
nmr600-vnmrs600
Archive directory:

Sample directory:

FidFile: 600ras062413.005

Pulse Sequence: CARBON (s2pul)
Solvent: cdcl3
Data collected on: Jun 24 2013

Temp. 25.0 C / 298.1 K
Operator: ras

Relax. delay 1.000 sec
Pulse 45.0 degrees
Acq. time 0.865 sec
Width 37878.8 Hz
112 repetitions
OBSERVE C13, 150.8132523 MHz
DECOUPLE H1, 599.7768382 MHz
Power 46 dB
continuously on
WALTZ-16 modulated
DATA PROCESSING
Line broadening 0.5 Hz
FT size 65536
Total time 129 hr, 31 min

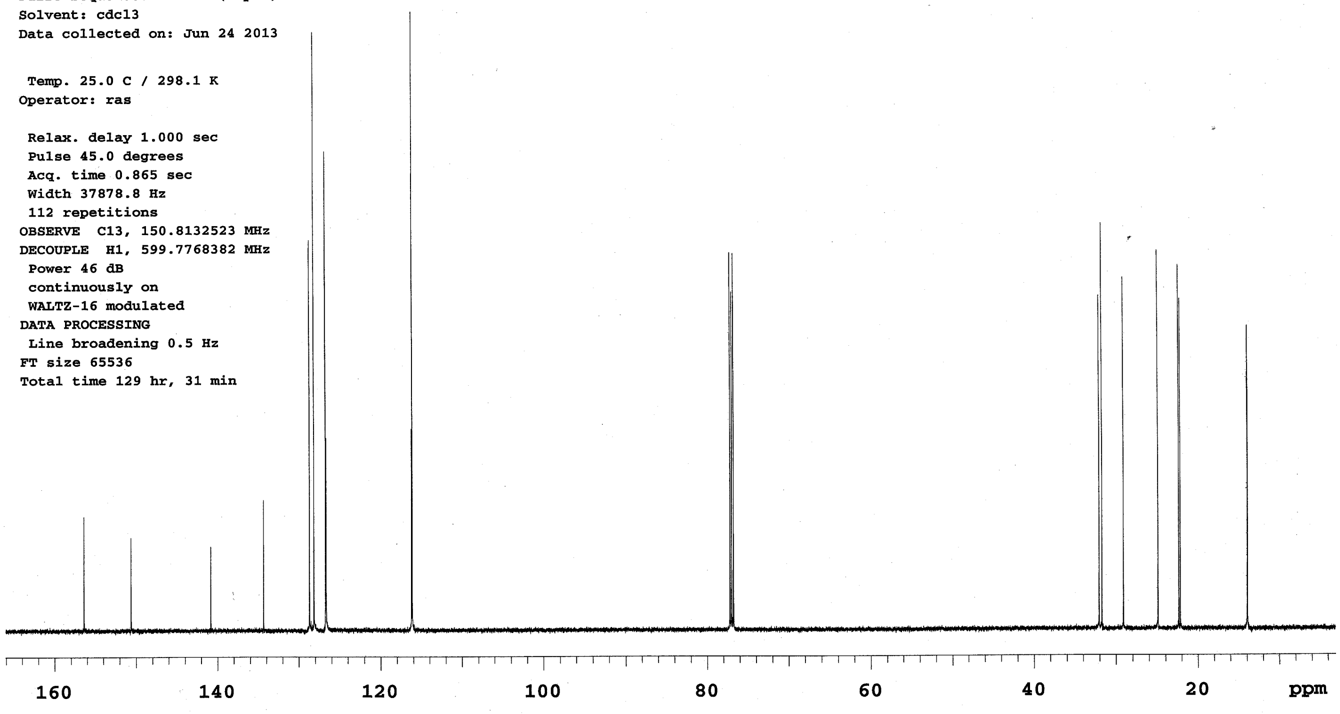

Supplement: File 1 — Detailed synthetic procedures for the synthesis of the arylgold compounds and vinyl ethers as well as NMR spectra for all new compounds. [file Beilstein_J_Org_Chem-09-2002-s001.pdf]
